# Bipotent transitional liver progenitor cells contribute to liver regeneration

**DOI:** 10.1038/s41588-023-01335-9

**Published:** 2023-03-13

**Authors:** Wenjuan Pu, Huan Zhu, Mingjun Zhang, Monika Pikiolek, Caner Ercan, Jie Li, Xiuzhen Huang, Ximeng Han, Zhenqian Zhang, Zan Lv, Yan Li, Kuo Liu, Lingjuan He, Xiuxiu Liu, Markus H. Heim, Luigi M. Terracciano, Jan S. Tchorz, Bin Zhou

**Affiliations:** 1 State Key Laboratory of Cell Biology, Shanghai Institute of Biochemistry and Cell Biology, Center for Excellence in Molecular Cell Science, Chinese Academy of Sciences, University of Chinese Academy of Sciences, Shanghai, China; 2 Novartis Institutes for BioMedical Research, Novartis Pharma AG, Basel, Switzerland; 3 Institute of Medical Genetics and Pathology, University Hospital Basel, Basel, Switzerland; 4 Key Laboratory of Systems Health Science of Zhejiang Province, School of Life Science, Hangzhou Institute for Advanced Study, University of Chinese Academy of Sciences, Hangzhou, China; 5 School of Life Sciences, Westlake University, Hangzhou, China; 6 Department of Biomedicine, University Hospital and University of Basel, Basel, Switzerland; 7 Clarunis University Center for Gastrointestinal and Liver Diseases, Basel, Switzerland; 8 Department of Biomedical Sciences, Humanitas University, Milan, Italy; 9 IRCCS Humanitas Research Hospital, Milan, Italy; 10 School of Life Science and Technology, ShanghaiTech University, Shanghai, China; 11 New Cornerstone Science Laboratory, Shenzhen, China

**Keywords:** Stem cells, Stem-cell research, Liver diseases

## Abstract

Following severe liver injury, when hepatocyte-mediated regeneration is impaired, biliary epithelial cells (BECs) can transdifferentiate into functional hepatocytes. However, the subset of BECs with such facultative tissue stem cell potential, as well as the mechanisms enabling transdifferentiation, remains elusive. Here we identify a transitional liver progenitor cell (TLPC), which originates from BECs and differentiates into hepatocytes during regeneration from severe liver injury. By applying a dual genetic lineage tracing approach, we specifically labeled TLPCs and found that they are bipotent, as they either differentiate into hepatocytes or re-adopt BEC fate. Mechanistically, Notch and Wnt/β-catenin signaling orchestrate BEC-to-TLPC and TLPC-to-hepatocyte conversions, respectively. Together, our study provides functional and mechanistic insights into transdifferentiation-assisted liver regeneration.

## Abstract (web summary)

Transitional liver progenitor cells (TLPCs), which derive from biliary epithelial cells (BECs), differentiate into hepatocytes after serious liver damage. Notch and WNT/β-catenin signaling regulate BEC-to-TLPC and TLPC-to-hepatocyte conversions, respectively.

## Main

The liver performs critical life-enabling metabolic, endocrine and secretory functions via its two epithelial cell compartments. Hepatocytes metabolize nutrients and xenobiotics, produce and recycle proteins, and generate bile acids. BECs (also termed cholangiocytes) constitute the bile duct network responsible for collecting and transporting bile into the gut, thereby supporting metabolite excretion and digestion^1,2^. Maintaining a functional hepatocyte pool is essential to guarantee liver function during homeostatic cell turnover or in response to injury^3–6^.

Previous genetic lineage tracing studies demonstrated that the hepatocyte pool is mainly replenished through self-renewal of pre-existing hepatocytes rather than differentiation from liver stem/progenitor cells during homeostasis and injury conditions leaving hepatocyte proliferation intact^7–11^. BECs are also able to proliferate and generate auxiliary biliary ducts in a regenerative process called ductular reaction^12^. However, when hepatocytes become senescent and hepatocyte-mediated liver regeneration is impaired in mice, BECs can serve as facultative liver progenitor cells (LPCs) and transdifferentiate into functional replication-competent hepatocytes^13–18^. In zebrafish, hepatic BECs or LPCs convert into hepatocytes after severe loss of hepatocytes^19,20^, in a process that is tightly modulated by genetic and epigenetic regulators to enable efficient liver regeneration^21–23^. Given the widespread hepatocyte senescence and impaired liver regeneration in patients with chronic liver disease and cirrhosis^24,25^, BEC-to-hepatocyte transdifferentiation could be an important repair mechanism in humans. Therapies promoting this transdifferentiation could open up a new treatment avenue to address this highly unmet medical need. However, the cellular identity, as well as the molecular mechanisms, promoting BEC-to-hepatocyte transdifferentiation during this important regenerative process remains elusive.

Here we generated a mouse model, in which the *fumarylacetoacetase* (*Fah*) gene is deleted, causing hepatocyte senescence during liver regeneration, modeling human hereditary tyrosinemia type I caused by a deficiency in FAH^26^ and inducing BEC-to-hepatocyte transdifferentiation. Combining single-cell RNA sequencing (scRNA-seq) with dual recombinase-mediated lineage tracing and pathway modulations, we identified a subset of BECs with LPC potential, as well as the mechanisms coordinating stepwise BEC-to-hepatocyte transdifferentiation.

## Results

### Generation of a model for BEC-to-hepatocyte conversion

**Extended Data Fig. 1:**
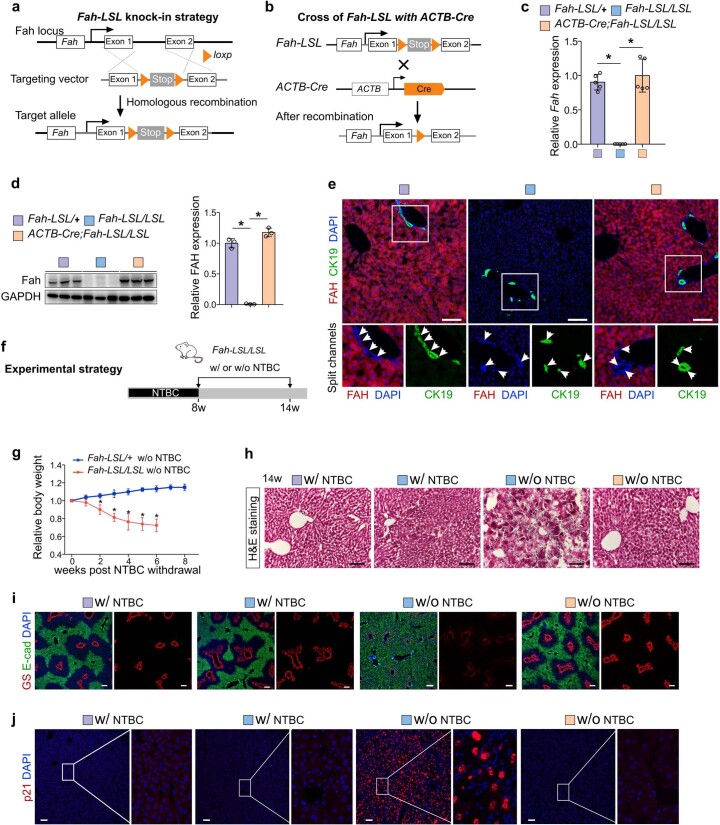
Generation and characterization of *Fah-LSL* mice. **a**, Schematic diagram showing the strategy for generation of *Fah-LSL* knock-in allele by homologous recombination using CRISPR/Cas9. **b**, Schematic diagram showing re-expression of *Fah* after LSL removal by crossing of *Fah-LSL* mice with *ACTB-Cre* mice. **c**, Relative mRNA expression levels of *Fah* in the livers of adult *Fah-LSL/* + , *Fah-LSL/LSL* and *Fah-LSL/LSL;ACTB-Cre* mice treated with NTBC. Data are the mean ± SD; n = 5 mice; *P < 0.0001, *P < 0.0001. **d**, Western blotting for FAH in the livers of adult *Fah-LSL/* + , *Fah-LSL/LSL* and *Fah-LSL/LSL;ACTB-Cre* mice treated with NTBC. Quantification of the relative protein levels of FAH was shown on the right. Data are the mean ± SD; n = 3 mice; *P < 0.0001, *P < 0.0001. Statistical analysis in c and d was performed by ANOVA followed by Bonferroni test for multiple comparisons and adjustments were made for multiple comparisons. **e**, Immunostaining for FAH and CK19 on the liver sections from adult *Fah-LSL/* + , *Fah-LSL/LSL*, and *Fah-LSL/LSL;ACTB-Cre* mice treated with NTBC. Arrowheads, CK19^+^FAH^–^ BECs. Scale bar, 100 µm. **f**, Schematic showing the experimental strategy for NTBC withdrawal (w/o NTBC) and tissue analysis at indicated time points. **g**, Weekly body weight measurements for *Fah-LSL/* + and *Fah-LSL/LSL* mice after NTBC withdrawal (w/o NTBC). The data are normalized to the body weights at week 0. Data represent mean ± SD; n = 5 mice; week2: *P < 0.0004; week3-wek6: *P < 0.0001. Statistical analysis was performed by multiple t test. Each row was analyzed individually without assuming a consistent SD. **h**, Hematoxylin and eosin (H&E) staining of liver sections. Scale bar, 100 µm. **i**,**j**, Immunostaining of GS and E-cad (i) and p21 (j) on liver sections from adult *Fah-LSL/* + , *Fah-LSL/LSL* and *Fah-LSL/LSL;ACTB-Cre* mice treated with NTBC (w/ NTBC) or at 8 weeks after NTBC removal (w/o NTBC). Scale bar, 100 µm.

We first generated a *Fah-LSL* mouse line, which contains a *Fah* deletion by introducing a LoxP-flanked Stop sequence (LSL) between exon1 and exon2, while allowing for Cre-induced *Fah* re-expression when needed (Extended Data Fig. 1a). Homozygous *Fah-LSL/LSL* mice lacked FAH expression and did not survive into adulthood without 2-(2-nitro-4-trifluoromethylbenzoyl)-1,3-cyclohexanedione (NTBC) treatment, a drug preventing injury in hepatocytes with FAH deletion^27^. In contrast, *Fah-LSL/*+mice and mice with Cre-LoxP-mediated removal of Stop sequence (*ACTB-Cre;Fah-LSL/Fah-LSL* mice), expressed FAH, were healthy and displayed normal growth (Extended Data Fig. 1b–e). We next analyzed the phenotypes in *Fah-LSL/LSL* mice after NTBC withdrawal. *Fah-LSL/LSL* mice were maintained with NTBC-containing water until 8 weeks of age. Compared to littermate *Fah-LSL/* + mice, *Fah-LSL/LSL* mice showed significant body weight loss at 2 weeks after NTBC withdrawal and were moribund within 8 weeks (Extended Data Fig. 1f,g). *Fah-LSL/LSL* mouse livers exhibited abnormal hepatic architecture, widespread injury throughout liver lobules, disrupted metabolic zonation and hepatocyte senescence (p21 staining) in virtually all hepatocytes (Extended Data Fig. 1h–j). These data demonstrate that our *Fah-LSL* mice recapitulate the common pathological phenotypes of *Fah*^*−/−*^ mice^28,29^, characterized by fulminant liver failure and impaired hepatocyte-mediated regeneration, with the advantage that our *Fah-LSL* knockout allele allows for Cre-induced restoration of *Fah* expression.

**Extended Data Fig. 2:**
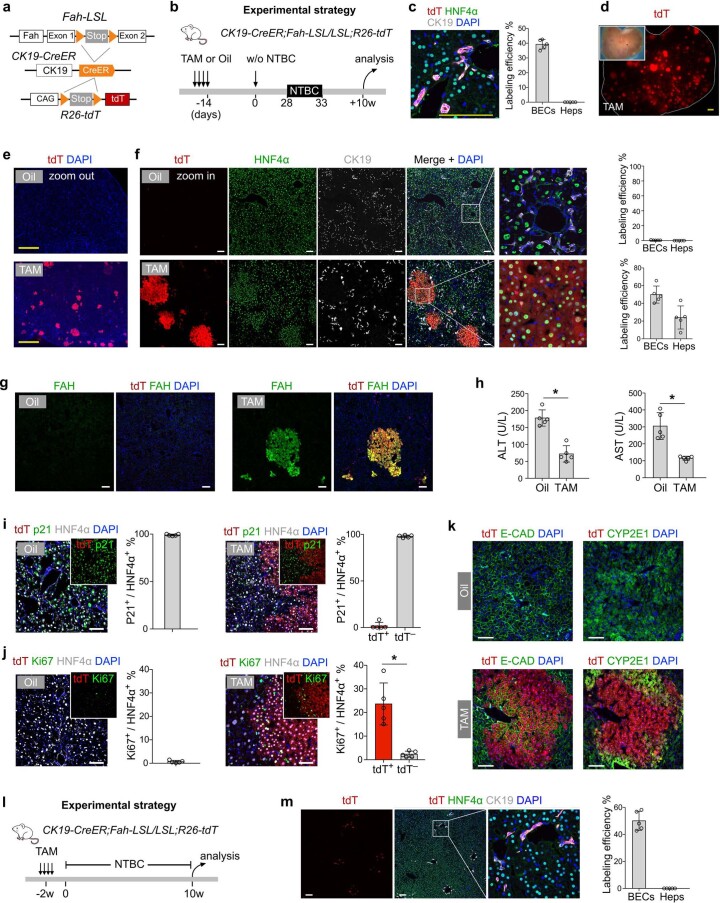
BEC-derived hepatocytes contribute to liver regeneration. **a**, Schematic diagram showing the experimental design for recovery of *Fah* gene in BECs and lineage tracing. **b**, Schematic diagram showing the experimental strategy of lineage tracing in *CK19-CreER;Fah-LSL/LSL;R26-tdT* mice. **c**, Immunostaining for tdT, CK19, and HNF4α on liver sections collected at day 0. Scale bar, 1 mm. Right panel shows quantification of cell labeling efficiency. Data represent mean ± SD; n = 5 mice. **d**, Whole-mount fluorescence liver images. Scale bars, 1 mm. **e**, Immunostaining for tdT on liver sections from mice treated with TAM or oil. Scale bars, 1 mm. **f**, Immunostaining for tdT, HNF4α, and CK19 on the liver sections from mice treated with TAM or oil. Quantification of percentage of tdT^+^ BECs and tdT^+^ Heps is shown in the adjacent graph. Data represent mean ± SD; n = 5 mice. Scale bars, 100 µm. **g**, Immunostaining for tdT and FAH on the liver sections from mice treated with TAM or oil. Scale bars, 100 µm. **h**, Serum ALT and AST of mice treated with TAM or oil. Data are the mean ± SD; n = 5 mice. *P < 0.0001; *P < 0.0001. **i**,**j**, Immunostaining for tdT, HNF4α and p21 (i) or Ki67 (j) on liver sections. Inserts show green and red fluorescence channels. Quantification of Ki67 or p21 staining in tdT^+^ and tdT^–^ hepatocytes is shown in the adjacent graph. Data represent mean ± SD; n = 5 mice. *P < 0.0001; Statistical analysis in h and j was performed by two-tailed unpaired Student’s *t* test. **k**, Immunostaining for tdT with E-CAD or CYP2E1 on the liver sections from mice treated with TAM or Oil. Scale bars, 100 µm. **l**, Schematic figure showing the experimental strategy. **m**, Immunostaining for tdT, CK19, and HNF4α on the liver sections. Data represent mean ± SD; n = 5 mice. Scale bars, 100 µm. W/o NTBC, without NTBC.

**Extended Data Fig. 3:**
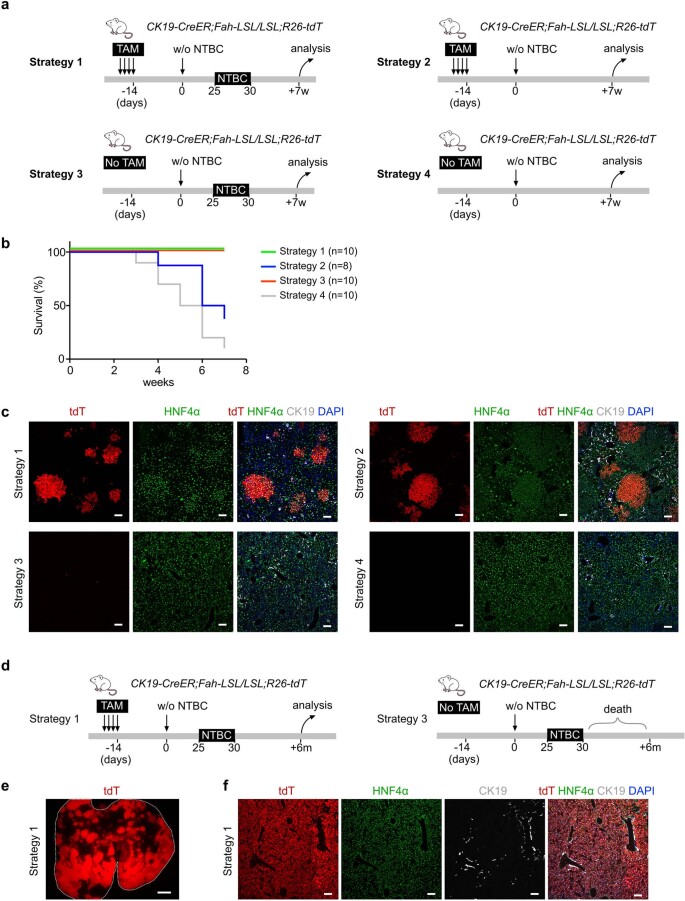
BECs-derived hepatocytes contribute to liver regeneration. **a**, Schematic showing experimental strategies. **b**, Kaplan-Meier plot showing survival of *CK19-CreER;Fah-LSL/LSL;R26-tdT* mice in different strategies. **c**, Immunostaining for tdT, CK19, and HNF4α on liver sections of indicated mice collected at week 7. Scale bars, 100 µm. **d**, Schematic showing experimental strategies. **e**, Whole-mount tdT fluorescent liver images from indicated mice at 6 months after the first NTBC removal. Scale bars, 2 mm. **f**, Immunostaining for tdT, CK19, and HNF4α on liver sections of indicated mice collected at month 6 (+6 m). Scale bars, 100 µm. W/o NTBC, without NTBC.

Next, we generated *CK19-CreER;Fah-LSL/LSL;R26-tdT* mice (Extended Data Fig. 2a), in which tdTomato (tdT) was induced and the *Fah* gene was restored in BECs after tamoxifen (TAM)-induced Cre-loxP recombination. *CK19-CreER* specifically targeted BECs but not hepatocytes^9,30^. We assessed the potential of BEC-to-hepatocyte transdifferentiation during liver regeneration in our injury model (Extended Data Fig. 2b). BECs (~40%) but not hepatocytes were specifically tdT-labeled before injury (Extended Data Fig. 2c). After the injury, we observed many round-shaped tdT^+^ clones in TAM-treated *CK19-CreER;Fah-LSL/LSL;R26-tdT* livers (Extended Data Fig. 2d), and tdT was detected in hepatocytes (~25%; Extended Data Fig. 2e,f). Of note, all tdT^+^ hepatocytes were positive for FAH in TAM-treated mouse livers (Extended Data Fig. 2g), which substantially reduced the severity of liver injury (Extended Data Fig. 2h). These tdT^+^ hepatocytes did not express p21 and showed increased proliferation when compared with tdT^–^ hepatocytes (Extended Data Fig. 2i,j). Large clones of tdT^+^ hepatocytes re-established metabolic zonation by expressing periportal and pericentral zonation markers in the respective lobular zones (Extended Data Fig. 2k). Of note, tdT^+^ hepatocytes only occurred in mice treated with TAM, and these BEC-derived hepatocytes substantially increased the long-term survival of mice in our injury model (Extended Data Fig. 3a,c–f). While this clearly supports that BEC-to-hepatocyte transdifferentiation promotes liver regeneration, short-term (5 d) NTBC reintroduction was necessary to ensure survival and enable this regenerative process to compensate for the fulminant liver failure in our model (Extended Data Fig. 3a,b). We did not detect any tdT^+^ hepatocytes in TAM-treated *CK19-CreER;Fah-LSL/LSL;R26-tdT* mice when NTBC was given throughout the experiment (Extended Data Fig. 2l,m), excluding potential ectopic activation of *CK19-CreER* in hepatocytes, and also suggesting that loss of FAH function and associated liver injury is necessary to induce BEC-to-hepatocyte transdifferentiation.

**Extended Data Fig. 4:**
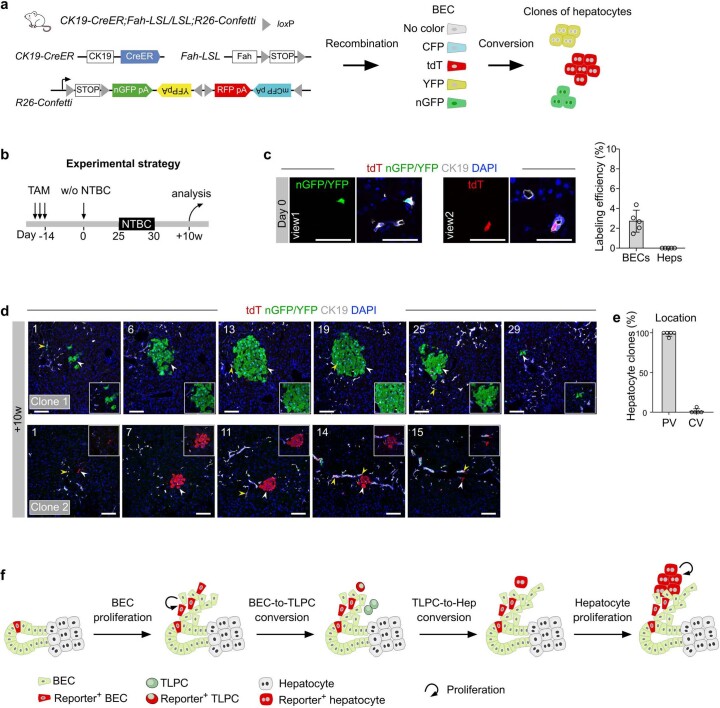
Clonal analysis of single BEC-derived cells. **a**, Schematic figure showing that single BECs were marked by either tdT, CFP, YFP or nGFP after TAM-induced recombination, as their conversion into hepatocytes in *CK19-CreER;Fah-LSL/LSL;R26-confetti* mice. **b**, Schematic figure showing the experimental strategy. **c**, Immunostaining for tdT, nGFP/YFP, and CK19 on the liver sections from *CK19-CreER;Fah-LSL/LSL;R26-confetti* mice. 2 exemplary images (view 1 + 2) from different portal tracts are shown. The percentage of reporter^+^ BECs and reporter^+^ hepatocytes are shown in the adjacent graph. Data are the mean ± SD; n = 5 mice. Scale bars, 50 µm. **d**, Immunostaining for tdT, nGFP/YFP, and CK19 on the liver sections collected from *CK19-CreER;Fah-LSL/LSL;R26-confetti* mice at week 10. Numbers in the images indicated the sequential order of serial 20 µm-sections of 2 entire clones. White arrowhead, reporter^+^ hepatocytes; yellow arrowhead, reporter^+^ BECs. Scale bars, 100 µm. **e**, Percentage of reporter^+^ hepatocyte clones located close to the PV or CV region. **f**, Cartoon image showing that BECs proliferate before conversion into TLPCs, and TLPCs converted into either hepatocytes or BECs. Single BECs could contribute to both hepatocytes and BECs during liver injury. Data are the mean ± SD; n = 5 mice. PV, portal vein; CV, central vein.

Furthermore, we crossed *CK19-CreER;Fah-LSL/LSL* with multicolor fluorescence reporter (*R26-Confetti* mice^31^) for clonal analysis of single BEC-derived cells during regeneration (Extended Data Fig. 4a). Due to sparse labeling of BECs with one of the four reporters in *CK19-CreER;Fah-LSL/LSL;R26-Confetti* mice, a single-color clone detected at the end of the experiment would be regarded as progeny from a single BEC (Extended Data Fig. 4a). While TAM treatment selectively labeled single BECs in livers at 0 weeks, we detected single-color hepatocyte clones, which were located near the portal veins but not central veins at 10 weeks (Extended Data Fig. 4b–e). Of note, a subset of single-color clones contained both BECs and hepatocytes, suggesting single BECs could give rise to both cell lineages over time (Extended Data Fig. 4d,f). Together, our *Fah-LSL* mice provide a new model for studying BEC-to-hepatocyte transdifferentiation.

### Identification of a liver progenitor cell state in severely injured livers

**Fig. 1:**
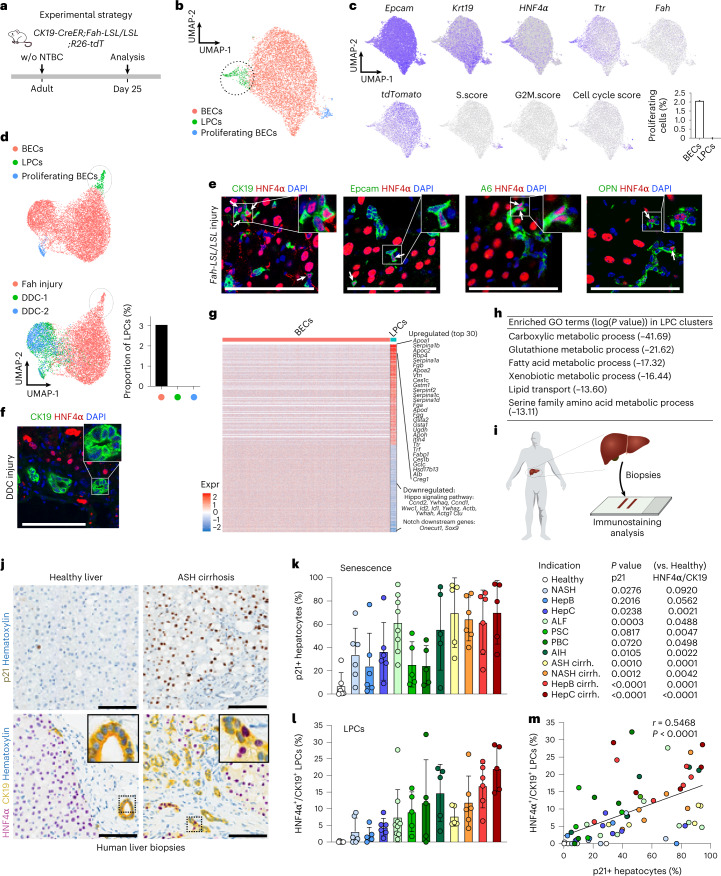
Identification of an LPC state in mouse and human livers. **a**, Schematic showing experimental strategy. **b**, UMAP visualization of the EPCAM^+^ epithelial cell clusters—BECs, LPCs and proliferating BECs. **c**, UMAP plots show expression of indicated genes and cell cycle score. Proportion of proliferating cells in BECs and LPCs is shown in the right panel. **d**, Integrated UMAP showing BECs from *Fah-LSL/LSL* mice and BECs from mice fed with DDC diet. DDC data are retrieved from previous studies—DDC-1 (ref. ^32^) and DDC-2 (ref. ^33^). Proportion of LPCs in three groups is shown in the right panel. **e**, Immunostaining for HNF4α, CK19, EPCAM, A6 and OPN on *Fah-LSL/LSL* liver sections. White arrows indicate LPCs. **f**, Immunostaining for CK19 and HNF4α liver sections from mice fed with DDC for 3 weeks. **g**, Heatmap showing the differentially expressed genes between BECs and LPCs. Each column represents a cell and each row represents a gene. **h**, Selected GO terms enriched in LPCs cluster. The gene enrichment analysis was done by Metascape, which uses the well-adopted hypergeometric test and Benjamini-Hochberg *P* value correction algorithm to identify all enriched ontology terms. **i**, Schematic showing experimental strategy. **j**, Immunostaining for p21, CK19 and HNF4α in the indicated human liver biopsies. **k**,**l**, Quantification of the percentage of hepatocytes with nuclear p21 staining (senescence, **k**) or CK19^+^ cells expressing HNF4α (LPCs, **l**). Data represent mean ± s.d. *n* = patients. In **k**, unpaired two-tailed *t* tests were used (versus healthy, *n* = 6): NASH (*n* = 6), hepatitis (Hep)B (*n* = 6), HepC (*n* = 6), acute liver failure (ALF; *n* = 8), PSC (*n* = 5), PBC; (*n* = 5), AIH (*n* = 5), ASH cirrhosis (*n* = 5), NASH cirrhosis (*n* = 6), HepB cirrhosis (*n* = 6), HepC cirrhosis (*n* = 5). In **l**, unpaired two-tailed *t* tests were used (versus healthy, *n* = 6): NASH (*n* = 6), HepB (*n* = 5), HepC (*n* = 6), ALF (*n* = 9), PSC (*n* = 5), PBC (*n* = 5), AIH (*n* = 5), ASH cirrhosis (*n* = 5), NASH cirrhosis (*n* = 6), HepB cirrhosis (*n* = 6), HepC cirrhosis (*n* = 5). **m**, Correlation plot of the LPC number and hepatocyte senescence percentage. Pearson correlation was performed for statistical analysis. Scale bars in all immunostaining images, 100 µm. Source data

**Extended Data Fig. 5:**
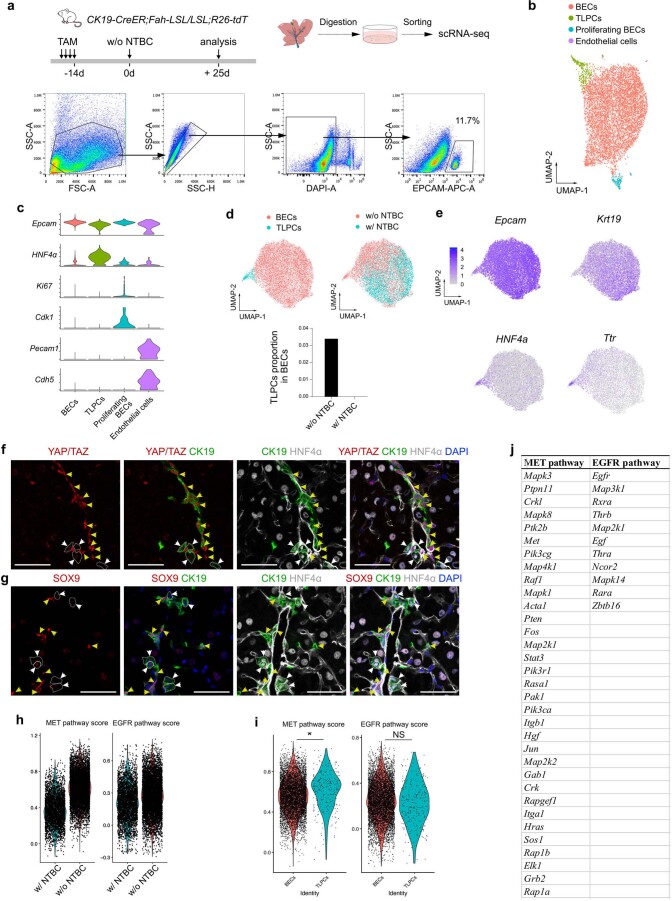
Analysis of gene expression in BECs and TLPCs. **a**, Schematic figure showing the experimental strategy and representative gating for FACS of EPCAM^+^ cells utilized for scRNA-seq. **b**, UMAP visualization of cells obtained from FACS sorting. **c**, Violin plots showing the marker gene expression levels per single cell in each cluster. **d**, Integrated UMAP showing BECs from *CK19-CreER;Fah-LSL/LSL;R26-tdT* mice kept with NTBC (w/ NTBC) and kept off NTBC for 25 days (w/o NTBC). Proportion of TLPCs in the two groups is shown. **e**, UMAP plots showing the expression of the indicated genes. **f**, Immunostaining for YAP/TAZ, CK19, and HNF4α on sections of liver collected at day 25 from *CK19-CreER;Fah-LSL/LSL;R26-tdT* mice. Scale bars, 50 µm. **g**, Immunostaining for SOX9, CK19, and HNF4α on sections of liver collected at day 25 from *CK19-CreER;Fah-LSL/LSL;R26-tdT* mice. Scale bars, 50 µm. Yellow arrowheads, BECs; white arrowheads, TLPCs. Each immunostaining image is representative of 5 mice. **h**, MET and EGFR pathway analysis by ssGSEA of EPCAM^+^ cells from d. **i**, MET and EGFR pathway analysis of BECs and TLPCs from mice (w/o NTBC) by ssGSEA. Mann-Whitney test was used to indentify the significant of the pathway scores between BECs and TLPCs. *p < 0.0001; NS, non-significant. **j**, List of genes with gene sets for MET and EGFR pathways.

Although BECs can transdifferentiate into hepatocytes following chronic liver injury and hepatocyte senescence^13–18^, the cellular identity of the BEC subset with LPC potential remains unclear. We, therefore, performed scRNA-seq of EPCAM^+^ cells under conditions allowing for BEC-to-hepatocyte transdifferentiation (25 d after NTBC withdrawal; Fig. 1a and Extended Data Fig. 5a) and compared our results with scRNA-seq data from EPCAM^+^ cells without conditions enabling such lineage conversion (short-term 3,5-diethoxycarbonyl-1,4-dihydrocollidine (DDC) diet-induced ductular reaction^32,33^). Uniform manifold approximation and projection (UMAP) identified several distinct clusters of EPCAM^+^ cells in *CK19-CreER;Fah-LSL/LSL* mice (Fig. 1b,c, Extended Data Fig. 5b,c and Supplementary Table 1). One subset was enriched for *Cdk1* and *Mki67* (proliferation markers), consistent with proliferating BECs within a ductular reaction that was also observed following DDC injury^32,33^ (Fig. 1d). Interestingly, we also identified a BEC subset that simultaneously expressed BEC markers (*Epcam* and *Ck19*) and hepatocyte markers (*Hnf4α* and *Ttr*; Fig. 1b,c and Extended Data Fig. 5b,c), which was not present in conditions without BEC-to-hepatocyte transdifferentiation (Fig. 1d). Given that this hybrid BEC-hepatocyte cluster was only present in mice with senescent hepatocytes and BEC-to-hepatocyte transdifferentiation, and because HNF4α is a master regulator inducing hepatocyte fate^34–36^, we defined this BEC subset as LPCs. Immunostaining for CK19, EPCAM, A6, OPN and HNF4α showed the appearance of LPCs in our injury model (Fig. 1e) but not in DDC-treated mice (Fig. 1f). Consistent with this, LPCs were also not detectable in uninjured livers of *Fah-LSL/LSL* mice that received NTBC (Extended Data Fig. 5d,e).

We next compared gene expression profiles of LPCs and BECs and found more than 400 genes differentially expressed between the two clusters (Fig. 1g and Supplementary Table 2). Among the top 30 upregulated genes in LPCs, there were several hepatocyte markers such as *Alb*, *Serpina1a*, *Hsd17b13* and *Apoh*. Moreover, gene set enrichment analysis (GSEA) revealed that most of the top-upregulated GO terms in LPCs were associated with hepatocyte functions, such as carboxylic metabolic process, glutathione metabolic process, fatty acid metabolic process, detoxification and lipid transport (Fig. 1h). Besides, we observed that several Hippo signaling pathway-related genes and Notch downstream genes, pathways conferring BEC identity^37–40^, were downregulated in the LPC cluster, such as *Id2*, *Id1*, *Clu*, *Onecut1* and *Sox9* (Fig. 1g). Moreover, immunostaining confirmed that CK19^+^HNF4α^+^ LPCs expressed lower YAP/TAZ and SOX9 levels compared with CK19^+^HNF4α^–^ BECs (Extended Data Fig. 5f,g). Additionally, *MET* and *EGFR* pathway genes were upregulated in BECs from mice with injured livers compared with control mice (Extended Data Fig. 5h), consistent with previous work^41^. In the injured liver, there was a higher MET pathway score in LPCs than BECs, while there was no difference in the EGFR pathway score between LPCs and BECs (Extended Data Fig. 5i,j).

**Extended Data Fig. 6:**
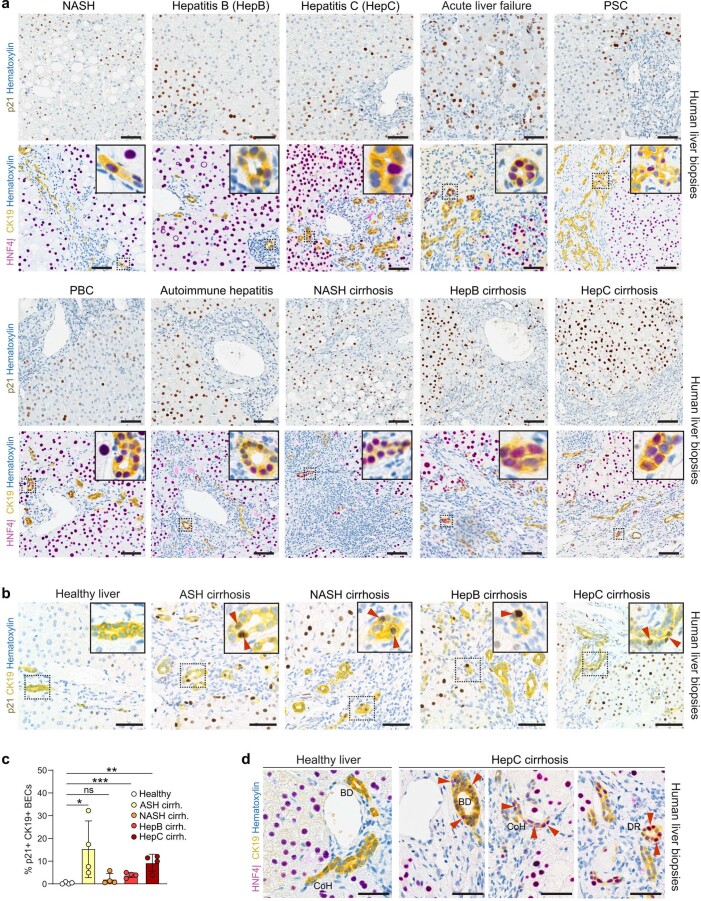
Presence of TLPCs and hepatocyte senescence in human livers. **a**, Immunostaining for p21 or HNF4α and CK19 in patient biopsies from the indicated human liver disease indications. Quantifications for p21^+^ hepatocytes and HNF4α^+^CK19^+^ TLPCs are shown in Fig. 1. Scale bars, 100 µm. **b**, Immunostaining for p21 and CK19 in the indicated human liver biopsies. Scale bars, 50 µm. **c**, Quantification of p21^+^ BECs in the indicated liver biopsies. Data represent mean ± SD. n = patients. Unpaired two-tailed *t* tests were used (vs. healthy, n = 4): alcoholic steatohepatitis (ASH) cirrhosis (n = 4, *P = 0.0495)): non-alcoholic steatohepatitis (NASH) cirrhosis (n = 4, *P* = 0.2207), Hepatitis (Hep)B cirrhosis (n = 4, ***P = 0.0009), HepC cirrhosis (n = 4, **P = 0.0031). **d**, Immunostaining showing HNF4α^+^CK19^+^ TLPCs within different BEC populations in the indicated biopsies (BD, bile duct; DR, ductular reaction: CoH, canals of Hering). Scale bars, 50 µm.

To assess whether CK19^+^HNF4α^+^ cells were also present in patients with severe liver injury, we analyzed biopsies from healthy livers and 11 different liver disease indications (Supplementary Table 3), including NASH and viral hepatitis with and without cirrhosis, primary biliary cirrhosis (PBC), primary sclerosing cholangitis (PSC), acute liver failure, autoimmune hepatitis (AIH) and alcoholic steatohepatitis (ASH) with cirrhosis (ASH cirrhosis). We observed significant numbers of p21^+^ hepatocytes in patients with ASH cirrhosis (Fig. 1i–k) and in the majority of other liver disease indications (Extended Data Fig. 6a), indicating substantial senescence, similar to what we observed in our animal model. In contrast to healthy livers, ASH cirrhosis patients (Fig. 1j,l) and most other patients with severe liver disease (Extended Data Fig. 6a) showed CK19^+^ cells with nuclear HNF4α staining. Interestingly, we found a positive correlation between hepatocyte senescence and CK19^+^HNF4α^+^ LPCs across 11 liver disease indications, with consistently higher percentages of both p21^+^ hepatocytes and LPCs in patients with cirrhosis compared to those with a non-cirrhotic milder form of the respective disease (Fig. 1j–m and Extended Data Fig. 6a). This suggests that LPCs are common in patients with senescent hepatocytes across multiple liver disease indications. Notably, the majority of BECs in cirrhotic livers did not express p21, supporting their potential to proliferate during a ductular reaction and to convert into functional hepatocytes via LPCs (Extended Data Fig. 6b,c). Interestingly, CK19^+^HNF4α^+^ BECs were not restricted to ductular reactions, but we also found them in canals of Hering and bile ducts of cirrhotic livers (Extended Data Fig. 6d).

### LPCs are a transitional cellular state between BECs and hepatocytes

**Fig. 2:**
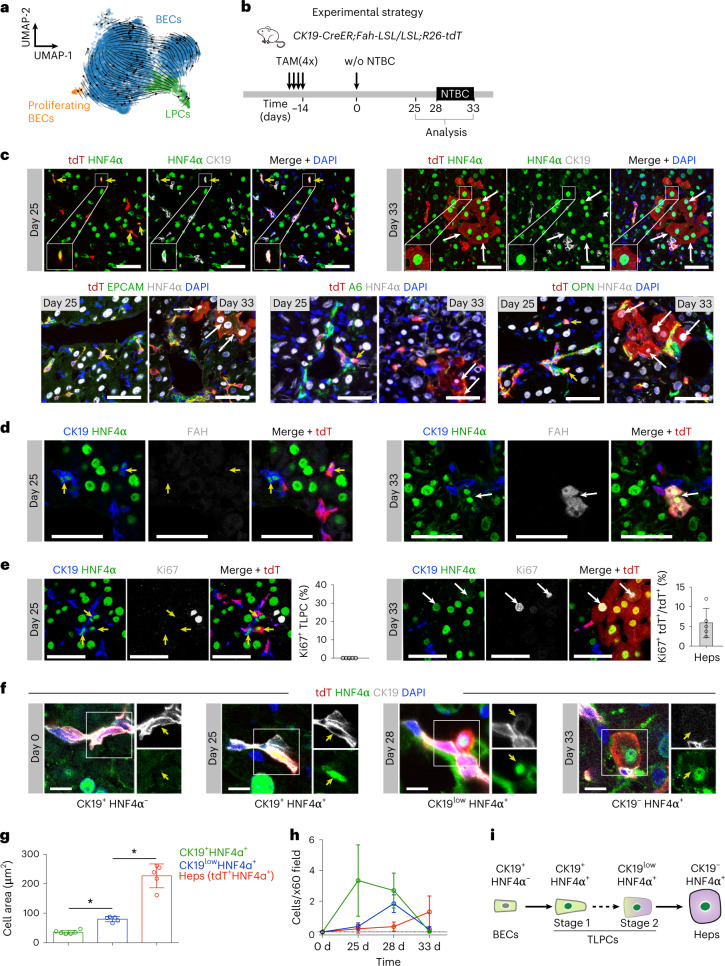
LPCs are a transitional cellular state between BECs and hepatocytes during transdifferentiation. **a**, UMAP embedding of RNA velocity of EPCAM^+^ cells collected from *Fah-LSL/LSL* mice at day 25 after initial NTBC removal indicates the transition from BECs to LPCs. **b**, Schematic showing experimental strategy. **c**, Immunostaining for tdT, HNF4α, CK19, EPCAM, A6 or OPN on liver sections collected at days 25 and 33 after NTBC removal from *CK19-CreER;Fah-LSL/LSL;R26-tdT* mice. Yellow arrows indicate tdT^+^ LPCs and white arrows indicate tdT^+^ hepatocytes. Scale bars, 100 µm. **d**, Immunostaining for tdT, HNF4α, CK19 and FAH on liver sections collected at days 25 and 33 after NTBC removal. Scale bars, 50 µm. Yellow arrows indicate LPCs and white arrows indicate tdT^+^ hepatocytes. **e**, Immunostaining of tdT, HNF4α, CK19 and Ki67 on liver sections collected at days 25 and 33 after NTBC removal. Yellow arrows indicate LPCs and white arrows indicate tdT^+^ hepatocytes. Percentage of Ki67^+^ TLPCs and Ki67^+^tdT^+^ hepatocytes in tdT^+^ hepatocytes is shown on the right panel. Data represent mean ± s.d.; *n* = 5 mice. Scale bars, 100 µm. **f**, Immunostaining for tdT, HNF4α and CK19 on liver sections collected at different time points. Scale bars, 10 µm. **g**, Cell area quantification in TLPCs (CK19^+^HNF4α^+^ and CK19^low^HNF4α^+^) and hepatocytes (tdT^+^). Data represent mean ± s.d.; CK19^+^HNF4α^+^: *n* = 6 mice, CK19^low^HNF4α^+^ and Heps: *n* = 5 mice; CK19^low^HNF4α^+^ versus CK19^+^HNF4α^+^: *P = 0.02; Heps versus CK19^low^HNF4α^+^: *P < 0.0001. Statistical analysis was performed by ANOVA followed by Tukey’s method for multiple comparisons, and adjustments were made for multiple comparisons. **h**, Quantification of the number of TLPCs (CK19^+^HNF4α^+^ and CK19^low^HNF4α^+^) and hepatocytes (tdT^+^) per portal region ×60 field at different time points. Data represent mean ± s.d.; *n* = 5 mice. **i**, Schematic showing that TLPCs originate from BECs and differentiate into hepatocytes. Hep, hepatocyte; w/o, without.

To further characterize LPCs and their dynamics in liver regeneration, we first analyzed scRNA-seq data using UMAP embedding of RNA velocity from isolated EPCAM^+^ cells in mice with injury-induced BEC-to-hepatocyte transdifferentiation (*Fah-LSL/LSL* mice 25 d after NTBC removal). The dynamic trajectories indicated a transition from BECs to LPCs (Fig. 2a). Using *CK19-CreER;Fah-LSL/LSL;R26-tdT* mouse model, we labeled BECs before injury and collected livers for analysis at 25 and 33 d after NTBC withdrawal (Fig. 2b). At day 25, we observed lineage-labeled (tdT^+^) LPCs expressing both BEC markers (CK19, EPCAM, A6 and OPN) and hepatocyte marker HNF4α (Fig. 2c), indicating that LPCs originated from BECs. At day 33, we barely found LPCs anymore but instead detected tdT^+^ hepatocytes (Fig. 2c), suggesting that LPCs were in a transient or transitional state during transdifferentiation. Therefore, we considered this LPC population to be transitional liver progenitor cells (TLPCs). CK19^+^HNF4α^+^ TLPCs did not acquire mature hepatocyte markers such as FAH at day 25, whereas tdT^+^ hepatocytes became positive for FAH at day 33 while no longer expressing CK19 (Fig. 2d). TLPCs were quiescent (Ki67-negative) at day 25 (Fig. 2e), which is consistent with our scRNA-seq data (Fig. 1b,c). In contrast, we found pronounced proliferation of tdT^+^ hepatocytes at day 33 (Fig. 2e), suggesting that transdifferentiated hepatocytes contributed to liver regeneration. Collectively, our data indicate that TLPCs transitionally emerged during BEC-to-hepatocyte transdifferentiation.

To further characterize this process, we analyzed gene expression profiles and numbers of lineage-labeled BECs, TLPCs and hepatocytes at different time points during transdifferentiation. We observed that lineage-labeled cells had distinct patterns of marker expression, CK19^+^HNF4α^–^ (BECs), CK19^+^HNF4α^+^ (TLPC, stage 1), CK19^low^HNF4α^+^ (TLPC, stage 2) and CK19^–^HNF4α^+^ (hepatocytes; Fig. 2f), suggesting continuous sequential transdifferentiation. Moreover, cell size gradually increased as TLPCs converted into hepatocytes (Fig. 2g). When quantifying cells of different lineages for their CK19/HNF4α expression, we found a substantial enrichment of CK19^+^HNF4α^+^ TLPCs on day 25, CK19^low^HNF4α^+^ TLPCs on day 28 and CK19^–^tdT^+^ hepatocytes on day 33 after NTBC withdrawal (Fig. 2h). Together, these data suggest a gradual BEC-to-hepatocyte transdifferentiation via a TLPC state, characterized by dynamic changes in lineage marker gene expression (Fig. 2i).

### Bipotent TLPCs differentiate into hepatocytes or BECs

**Fig. 3:**
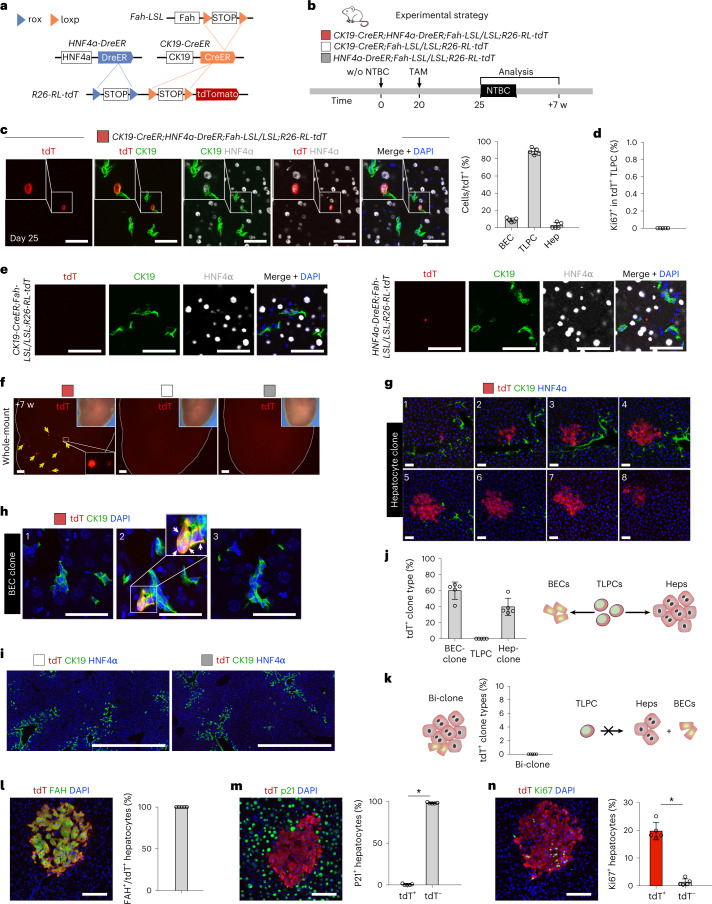
Bipotent TLPCs generate hepatocytes or adopt BEC fate during liver repair. **a**, Schematic showing the strategy for TLPC lineage tracing. **b**, Schematic showing experimental strategy. **c**, Immunostaining for tdT, HNF4α and CK19 on liver sections collected at day 25 from *CK19-CreER;HNF4α-DreER;Fah-LSL/LSL;R26-RL-tdT* mice. Scale bars, 50 µm. Right panel shows the percentage of BECs, TLPCs and hepatocytes in tdT^+^ cells. Data represent mean ± s.d.; *n* = 5 mice; total 289 tdT^+^ cells were counted (BECs: 25; TLPCs: 258; hepatocyte: 6). **d**, Percentage of Ki67^+^ cells in tdT^+^ TLPCs. Data represent mean ± s.d.; *n* = 5 mice. **e**, Immunostaining for tdT, HNF4α and CK19 on liver sections collected at day 25 from *CK19-CreER; Fah-LSL/LSL;R26-RL-tdT* or *HNF4α-DreER;Fah-LSL/LSL;R26-RL-tdT* mice. Scale bars, 50 µm. **f**, Whole-mount tdT fluorescent liver images from indicated mice at 7 weeks after the first NTBC removal. Bright field images are shown as inserts. Arrows indicate tdT^+^ clones. Scale bars, 1 mm. **g**, Immunostaining for tdT, HNF4α and CK19 on serial sections (20 µm) of livers collected at week 7 from *CK19-CreER;HNF4α-DreER;Fah-LSL/LSL;R26-RL-tdT* mice. Scale bars, 50 µm. **h**, Immunostaining of tdT, and CK19 on serial liver sections (20 µm) collected at week 7 from *CK19-CreER;HNF4α-DreER;Fah-LSL/LSL;R26-RL-tdT* mice. Scale bars, 50 µm. White arrows indicate tdT^+^ BECs. **i**, Immunostaining for tdT, HNF4α and CK19 on liver sections collected from *CK19-CreER;Fah-LSL/LSL;R26-RL-tdT* or *HNF4α-DreER;Fah-LSL/LSL;R26-RL-tdT* mice. Scale bars, 1 mm. **j**, Percentage of tdT^+^ clones containing BECs, TLPCs and hepatocytes. Schematic on the right panel showing that TLPCs could give rise to hepatocytes and revert back to BECs. Data represent mean ± s.d.; *n* = 5 mice; total 480 tdT^+^ clones were counted. **k**, Percentage of hybrid clone that consists of BECs and hepatocytes. Schematic showing that TLPCs could not give rise to hepatocytes and BEC simultaneously. Data represent mean ± s.d.; *n* = 5 mice. **l**–**n**, Immunostaining for tdT, FAH, p21 and Ki67 on tissue sections. Right panels are quantifications for percentage of hepatocytes expressing these markers. Scale bars, 100 µm. Data represent mean ± s.d.; *n* = 5 mice; **m**, *P < 0.0001; **n**, *P < 0.0001. Statistical analysis was performed by two-tailed unpaired Student’s *t* test in **m** and **n**. w, weeks.

To validate that CK19^+^HNF4a^+^ TLPCs are facultative LPCs with lineage conversion potential, we developed a Cre-loxP and Dre-rox-based dual genetic lineage tracing approach to indelibly label CK19^+^HNF4α^+^ TLPCs. Therefore, we generated *CK19-CreER;HNF4α-DreER;Fah-LSL/LSL;R26-RL-tdT* mice, in which tdT could be only expressed after TAM-induced removal of two Stop sequences by both Dre-rox (HNF4α^+^) and Cre-loxP (CK19^+^) recombinations (Fig. 3a). We treated mice with a low dosage of TAM at day 20 after NTBC removal and collected livers at day 25 and week 7. *CK19-CreER;Fah-LSL/LSL;R26-RL-tdT* or *HNF4α-DreER;Fah-LSL/LSL;R26-RL-tdT* mice were used as controls (Fig. 3b). Consistent with the small subset of CK19^+^HNF4α^+^ TLPCs we previously observed among BECs (Fig. 1b), we found a few tdT^+^ cells scattered in the portal tract region at day 25. We found 88.61 ± 3.80% of tdT^+^ cells were TLPCs (CK19^+^HNF4α^+^), 8.91 ± 2.19% of tdT^+^ cells were BECs (CK19^+^HNF4α^–^) and 2.48 ± 3.48% of tdT^+^ cells were hepatocytes (CK19^–^/HNF4α^+^) in *CK19-CreER;HNF4α-DreER;Fah-LSL/LSL;R26-RL-tdT* mouse livers (Fig. 3c). Of note, none of tdT^+^ TLPCs were Ki67^+^ (Fig. 3d), confirming TLPC quiescence. We did not detect any tdT^+^ cells in *CK19-CreER;Fah-LSL/LSL;R26-RL-tdT* or *HNF4α-DreER;Fah-LSL/LSL;R26-RL-tdT* control mouse livers at day 25 (Fig. 3e), confirming that *R26-RL-tdT* could only be activated by dual recombination.

We next assessed the lineage conversion potential of individual TLPCs over time at 7 weeks post low-dose TAM, enabling clonal analysis. We observed sporadic tdT^+^ clones in TAM-treated *CK19-CreER;HNF4α-DreER;Fah-LSL/LSL;R26-RL-tdT* mouse livers, but not in control mouse livers (Fig. 3f). tdT^+^ clones consisted of either hepatocytes (CK19^–^HNF4α^+^) or BECs (CK19^+^HNF4α^−^), whereas we did not find tdT^+^ TLPCs (CK19^+^HNF4α^+^) anymore (Fig. 3g,h). No tdT^+^ cells were detected in control liver sections (Fig. 3i). 60.14 ± 10.80% of the tdT^+^ clones were BEC clones and 39.86 ± 10.80% of the tdT^+^ clones were hepatocyte clones (Fig. 3j), suggesting that TLPCs contribute to BEC or hepatocyte lineages during liver regeneration. While TLPCs are bipotent, none of the individual tdT^+^ clones consisted of both hepatocytes and BECs (Fig. 3k), further suggesting that TLPCs did not proliferate in their bipotential state. Of note, all tdT^+^ hepatocytes were positive for FAH (Fig. 3l). tdT^+^ hepatocytes did not express p21 and showed significantly higher proliferation rates than tdT^–^ hepatocytes (Fig. 3m,n), suggesting their potential to repopulate the injured liver. Collectively, these data indicate that bipotent TLPCs can either transdifferentiate into hepatocytes or redifferentiate into BECs during liver regeneration.

### Notch signaling suppresses the differentiation of BECs into TLPCs

**Fig. 4:**
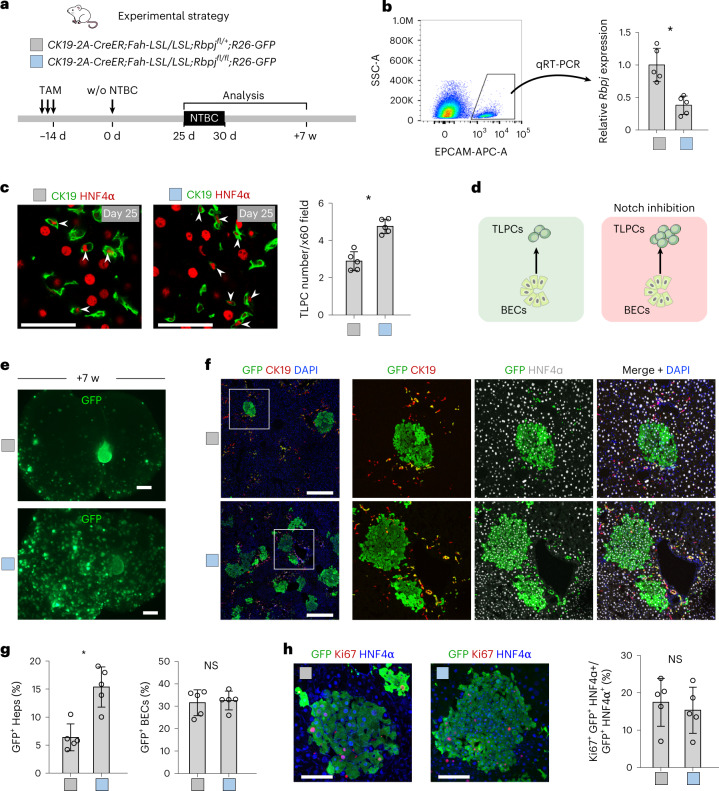
Inhibition of Notch signaling promotes the activation of TLPCs. **a**, Schematic figure showing experimental strategy for *Rbpj* gene knockout in BECs. **b**, Representative gating for FACS sorting of EPCAM^+^ BECs used for qRT-PCR. Relative expression levels of *Rbpj* mRNA in BECs from indicated mice. Data represent mean ± s.d.; *n* = 5 mice; *P = 0.0016. **c**, Immunostaining for HNF4α and CK19 on liver sections collected at day 25 after NTBC removal from indicated mice. Scale bars, 50 µm. Quantification of the number of TLPCs per portal region ×60 field is shown on the right panel. Data represent mean ± s.d.; *n* = 5 mice; *P = 0.0002. Total count of TLPCs in control: 717; in mutant: 1,159. White arrowheads indicate TLPCs. **d**, Schematic showing that inhibition of Notch signaling promotes BEC-to-TLPC transition. **e**, Whole-mount GFP fluorescent liver images collected at week 7 after NTBC removal. Scale bars, 2 mm. **f**, Immunostaining for GFP, HNF4α and CK19 on the liver sections collected at week 7 after NTBC removal. Scale bars, 500 µm. **g**, Percentage of GFP^+^ hepatocytes and GFP^+^ BECs. Data represent mean ± s.d.; *n* = 5 mice; GFP^+^ Heps (%): *P = 0.0016; GFP^+^ BECs (%): *P = 0.7691. **h**, Immunostaining for GFP, HNF4α and Ki67 on liver sections collected at week 7 after NTBC removal. Quantification of proliferation (Ki67) in GFP^+^ hepatocytes is shown on the right panel. Data represent mean ± s.d.; *n* = 5 mice. Scale bars, 100 µm. Statistical analysis was performed by two-tailed unpaired Student’s *t* test in **b**, **c**, **g** and **h**. NS, not significant; w, weeks.

**Extended Data Fig. 7:**
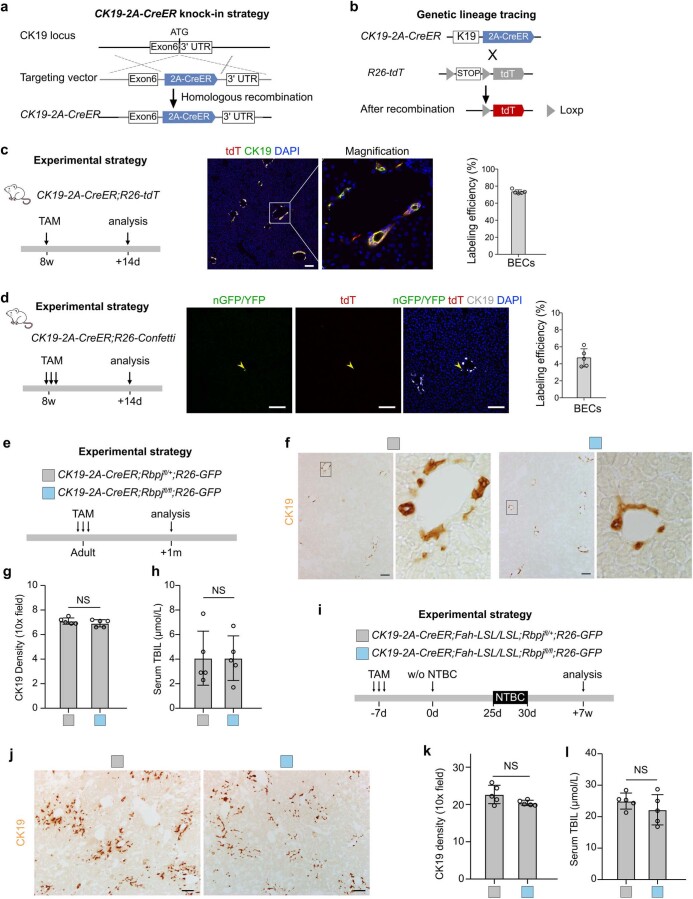
Generation of *CK19-2A-CreER* mouse line and *Rbpj* knockout in BECs. **a**, Schematic figure showing knock-in strategy for generation of the *CK19-2A-CreER* allele by homologous recombination. **b**, Genetic lineage tracing for CK19^+^ BECs by Cre-loxp recombination. **c**, Schematic figure showing the experimental strategy (left panel). Immunostaining for tdT and CK19 on liver section (middle panel). Quantification of percentage of BECs expressing tdT is shown in the adjacent graph. Data are the mean ± SD; n = 5 mice. **d**, Schematic figure showing the experimental strategy. Immunostaining for nGFP/YFP, tdT, and CK19 on liver sections. Quantification of percentage of reporter^+^ BECs is shown in the adjacent graph. Data represent mean ± SD; n = 5 mice. **e**,**i**, Schematic figures showing the experiment strategy. **f**,**j**, Immunostaining for CK19 on liver sections. **g**,**k**, Quantification of CK19 density per 10x field. Data represent mean ± SD; n = 5 mice; NS, non-significant. **h**,**l**, Quantification of serum total bilirubin (TBIL) from indicated mice. Statistical analysis was performed by two-tailed unpaired Student’s *t* test in g, h, k, l. Data represent mean ± SD; n = 5 mice; NS, non-significant. Scale bars, 100 µm.

**Extended Data Fig. 8:**
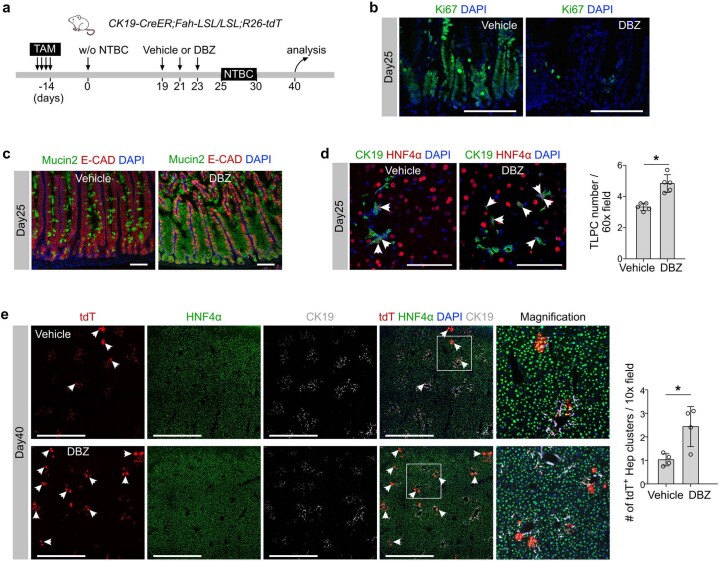
Inhibition of Notch signaling increases generation of TLPCs. **a**, Schematic figure showing the experimental strategy. Dibenzazepine (DBZ) is a pharmacologic Notch inhibitor, which inhibits Notch cleavage and blocks its activation. **b**, Immunostaining for Ki67 on intestine sections collected at day 25. Scale bars, 100 µm. **c**, Immunostaining for Mucin2 and E-CAD on small intestine sections collected at day 25. Scale bars, 100 µm. **d**, Immunostaining for CK19 and HNF4α on liver sections collected at day 25. Arrowheads, CK19^+^HNF4α^+^ TLPCs. Scale bars, 100 µm. Quantification of the number of TLPCs per 60x field in portal region is shown in the adjacent graph. Data are the mean ± SD; n = 5 mice. *P = 0.0006. **e**, Immunostaining for tdT, HNF4α, and CK19 on sections of liver collected at day 40. Number of tdT^+^ hepatocyte clusters per 10x field is quantified in the adjacent graph. Arrowheads, tdT^+^HNF4α^+^ hepatocyte clones. Data are the mean ± SD; n = 4 mice; *P = 0.0192. Statistical analysis was performed by two-tailed unpaired Student’s *t* test in d,e. Scale bars, 100 µm.

GO term analysis of scRNA-seq data and immunostaining data from *CK19-CreER;Fah-LSL/LSL* mice showed that Notch target genes, such as *Onecut1* and *Sox9*, were substantially reduced in TLPCs compared with BECs (Fig. 1g and Extended Data Fig. 5g), suggesting reduced Notch activity may promote the activation of TLPCs. To test this hypothesis, we blocked Notch signaling via *Rbpj* deletion in BECs and simultaneously traced these cells in our liver injury model, using *CK19-2A-CreER* mice that provide high recombination efficiency in BECs (Extended Data Fig. 7a–d). *CK19-2A-CreER;Fah-LSL-LSL;R26-GFP;Rbpj*^*fl/fl*^ (*Rbpj*^*fl/fl*^) mice enabled TAM-induced *Rbpj* deletion and GFP reporter expression in BECs, whereas *CK19-2A-CreER;Fah-LSL-LSL;R26-GFP;Rbpj*^*fl/+*^ (*Rbpj*^*fl/+*^) mice (enabling GFP expression in BECs while leaving one *Rbpj* allele intact) were used as controls (Fig. 4a). *Rbpj* expression was substantially reduced in BECs of *Rbpj*^*fl/fl*^ mice when compared with littermate *Rbpj*^*fl/+*^ control mice (Fig. 4b). We only observed an insignificant decrease in ductular reaction and comparable serum total bilirubin in BEC-specific *Rbpj* knockout mice compared with littermate controls (Extended Data Fig. 7e–l). This suggests that the inducible *Rbpj* deletion, in only the subset of BECs we traced, did not impair bile duct integrity as reported during developmental *Rbpj* deletion^42^. A comparable percentage of GFP^+^ BECs between the *Rbpj*^*fl/fl*^ and *Rbpj*^*fl/+*^ groups (Fig. 4g) indicates functional ductular reaction in mice with liver-specific Rbpj deletion^43^. TLPC numbers were significantly increased at day 25 (Fig. 4c,d), followed by increased numbers of GFP^+^ BEC-derived hepatocytes at week 7 post-NTBC removal in *Rbpj*^*fl/fl*^ mice compared with *Rbpj*^*fl/+*^ mice (Fig. 4e–g). Proliferation of GFP^+^ hepatocytes was comparable between *Rbpj*^*fl/fl*^ and *Rbpj*^*fl/+*^ mice (Fig. 4h). Similarly, mice treated with Notch inhibitor DBZ showed increased numbers of TLPCs and hepatocyte clones in *CK19-CreER;Fah-LSL/LSL;R26-tdT* mice (Extended Data Fig. 8). These data suggest that loss of Notch signaling promotes BEC-to-TLPC activation, therefore increasing BEC-to-hepatocyte conversion.

**Fig. 5:**
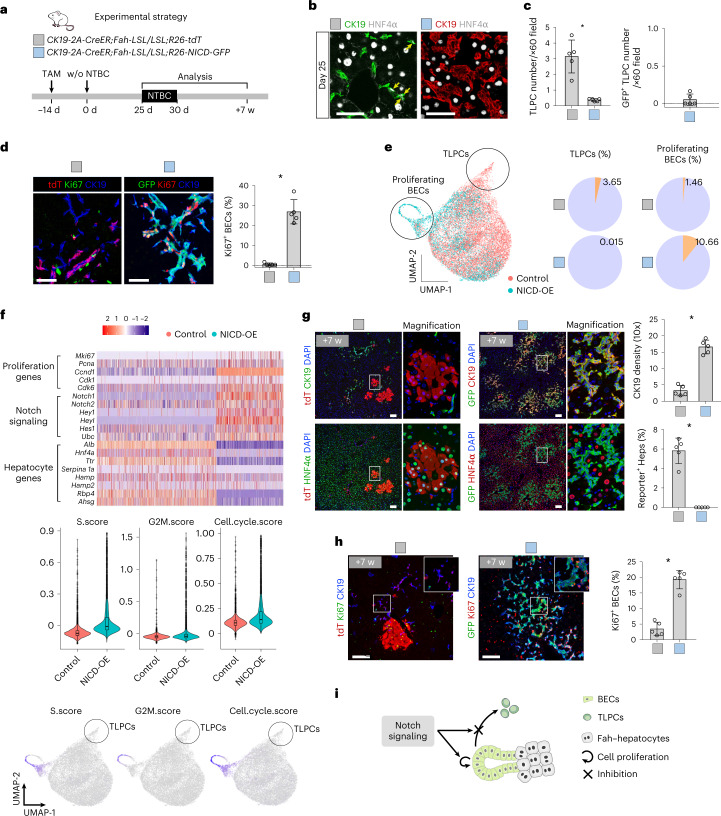
Notch activation inhibits BEC-to-TLPC activation. **a**, Schematic showing experimental strategy. Control, C*K19-2A-CreER;Fah-LSL/LSL;R26-tdT*. NICD-OE*, CK19-2A-CreER;Fah-LSL/LSL;R26-NICD-GFP*. **b**, Immunostaining for HNF4α and CK19 on liver sections collected at day 25 after NTBC removal. Scale bars, 50 µm. **c**, Quantification of the number of TLPCs and GFP^+^ TLPCs per portal region ×60 field from the indicated mice. Data represent mean ± s.d.; *n* = 5 mice; *P = 0.0004; Total count of TLPCs in control: 789, in NICD group: 92. **d**, Immunostaining for tdT or GFP, Ki67 and CK19 on liver sections collected at day 25 after NTBC removal. Percentage of Ki67^+^ BECs is shown on the right panel. Data represent mean ± s.d.; *n* = 5 mice; *P < 0.0001. Scale bars, 50 µm. **e**, UMAP visualization of EPCAM^+^ cells collected from control and NICD-OE mice at day 25 after NTBC removal. The percentage of TLPCs and proliferating BECs is shown on the right. **f**, Upper panel is scRNA-seq heatmap for EPCAM^+^ cells from control and NICD-OE mice showing expression of selected genes per cell. Middle panel shows violin plot of cell cycle score of genes related to G2M and S phases. Lower panel shows UMAP plot of gene set score of S, G2M and cell cycle. **g**, Immunostaining for tdT or GFP, HNF4α, and CK19 on liver sections collected at week 7 after NTBC removal. Quantification of the ductular reaction per ×10 field and the percentage of tdT^+^ or GFP^+^ hepatocytes is shown on the right panel. Data represent mean ± s.d.; *n* = 5 mice; CK19 density (10×): *P < 0.0001; Reporter^+^ Heps: *P < 0.0001. Scale bars, 100 µm. **h**, Immunostaining for tdT or GFP, Ki67 and CK19 on liver sections collected at week 7 after NTBC removal. Percentage of Ki67^+^ BECs is shown on the right panel^.^ Data represent mean ± s.d.; *n* = 5 mice; *P < 0.0001. Scale bars, 100 µm. **i**, Schematic showing that Notch signaling inhibited BEC-to-TLPCS activation. Statistical analysis was performed by two-tailed unpaired Student’s *t* test in **c**, **d**, **g** and **h**. w, weeks.

**Extended Data Fig. 9:**
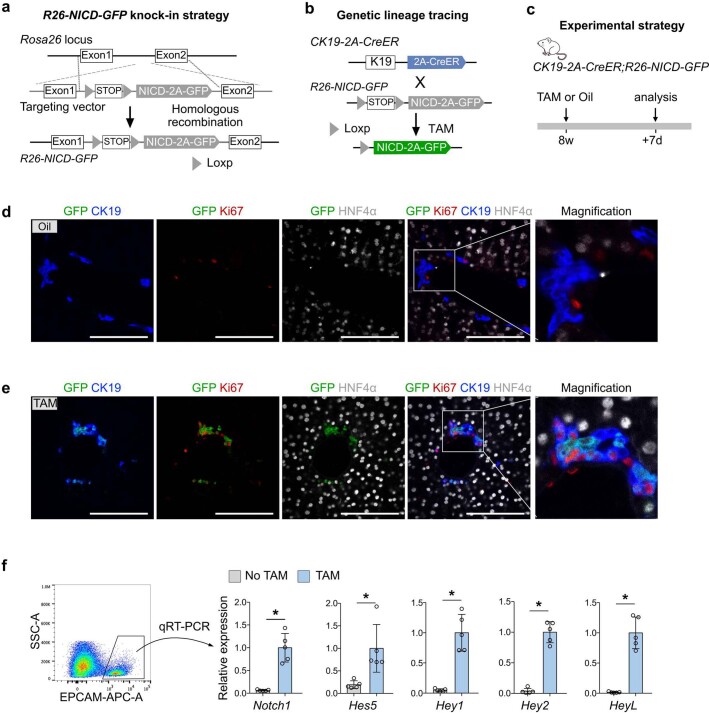
Over-expression of NICD and lineage tracing in BECs. **a**, Schematic figure showing the knock-in strategy for *R26-NICD-GFP* allele by homologous recombination using CRISPR/Cas9. **b**, Schematic figure showing the experimental strategy for over-expression of the Notch intracellular domain (NICD; activated Notch) in BECs by crossing *CK19-2A-CreER* with *R26-NICD-GFP* mice to generate *CK19-2A-CreER*;*R26-NICD-GFP* mice. **c**, Schematic showing the experimental strategy for TAM or oil treatment and tissue analysis at indicated time points. **d**,**e**, Immunostaining for GFP, CK19, Ki67 and HNF4α on liver sections from *CK19-2A-CreER;R26-NICD-GFP* mice treated with oil (d) or TAM (e). Scale bars, 100 µm. **f**, Isolation of EPCAM^+^ cells by flow cytometry followed by quantitative RT-PCR (qRT-PCR) analysis for expression of Notch1 and Notch target genes. Statistical analysis was performed by two-tailed unpaired Student’s *t* test in f. Data represent mean ± SD; n = 5 mice per group. *P = 0.0002 (*Notch1*); *P = 0.0099 (*Hes5*); *P = 0.0001 (*Hey1*); *P < 0.0001 (*Hey2*); *P < 0.0001 (*HeyL*).

To assess whether activation of Notch signaling would inhibit BEC-to-TLPC activation, we generated *R26-NICD-GFP* mice, in which Cre recombinase leads to the co-expression of the dominant active Notch intracellular domain (NICD) and GFP (Extended Data Fig. 9a,b). After crossing with *CK19-2A-CreER* mice, TAM treatment induced simultaneous GFP expression and Notch pathway activation in BECs (Extended Data Fig. 9c–f). Next, we generated *CK19-2A-CreER;Fah-LSL-LSL;R26-NICD-GFP* (NICD overexpression, NICD-OE) mice and *CK19-2A-CreER;Fah-LSL-LSL;R26-tdT* control mice, and collected livers at day 25 after NTBC removal for analysis (Fig. 5a). The number of TLPCs was significantly reduced following NICD overexpression, whereas BEC density and proliferation was dramatically increased (Fig. 5b–h and Supplementary Table 1). EPCAM^+^ cells sorted from NICD-OE livers revealed substantial enrichment for genes representative of Notch signaling and gene signatures indicating cell proliferation, and substantially decreased expression of hepatocyte genes, compared with EPCAM^+^ cells sorted from control mice (Fig. 5f and Supplementary Table 4). We did not detect any GFP^+^ hepatocytes at 7 weeks after NTBC removal in NICD-OE mice, compared with a considerable number of tdT^+^ hepatocytes in the control mice (Fig. 5g). Taken together, activation of Notch signaling suppresses BEC-to-TLPC induction and promotes BEC proliferation in injured livers (Fig. 5i).

### WNT/β-catenin signaling promotes TLPC-to-hepatocyte conversion

**Fig. 6:**
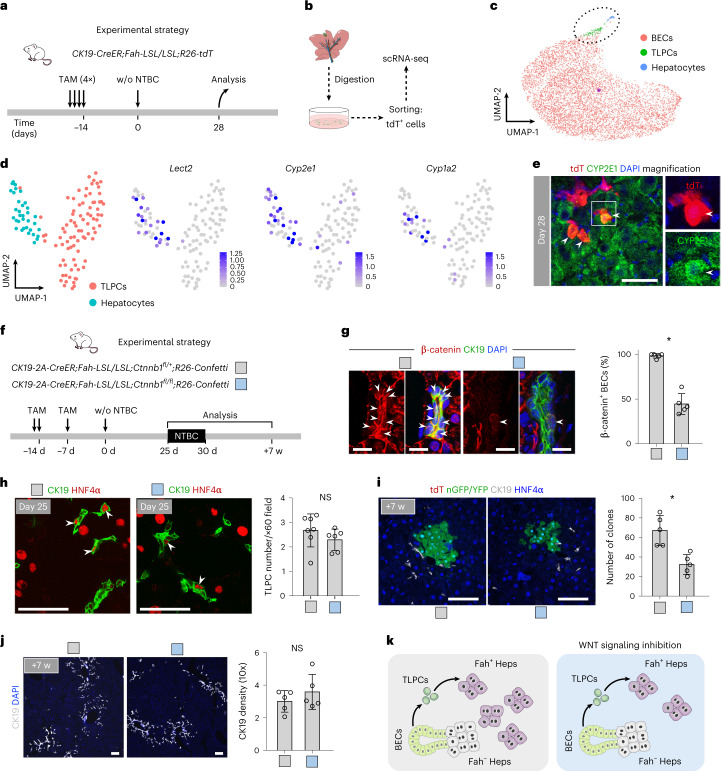
WNT signaling abrogation inhibits the conversion of TLPCs into hepatocytes. **a**, Schematic figure showing experimental strategy. **b**, Schematic figure showing strategy for sorting of tdT^+^ cells used for scRNA-seq. **c**, UMAP visualization of the cell populations collected from *CK19-CreER;Fah-LSL/LSL;R26-tdT* mice at day 28 after NTBC removal. **d**, UMAP visualization of TLPC and hepatocyte populations and UMAP plots show the expression of indicated genes in TLPC and hepatocyte populations. **e**, Immunostaining for tdT and CYP2E1 on the liver sections collected on day 28 after NTBC removal. Arrowheads indicate tdT^+^CYP2E1^+^ hepatocytes. Scale bars, 50 µm. **f**, Schematic showing experimental strategy for *Ctnnb1* gene knockout in BECs. **g**, Immunostaining for CK19 and β-catenin on liver sections. Percentage of β-catenin^+^ BECs. Data represent mean ± s.d.; *n* = 5 mice; *P < 0.0001. Scale bars, 10 µm. White arrowheads indicate β-catenin^+^ BECs. **h**, Immunostaining for CK19 and HNF4α on liver sections collected at day 25 after NTBC removal. Scale bars, 50 µm. Quantification of the number of TLPCs per portal region ×60 field is shown on the right panel. Data represent mean ± s.d.; *n* = 6–7 mice (control group: *n* = 7 mice, Ctnnb1 knockout group: *n* = 6 mice). Total count of TLPCs in control: 1,870; in Ctnnb1 knockout group: 1380. White arrowheads indicate TLPCs. **i**, Immunosta**i**ning for tdT, nGFP or YFP, CK19 and HNF4α on liver sections collected at week 7 after NTBC removal. The number of reporter^+^ clones per left liver lobe is shown on the right panel. Data represent mean ± s.d.; *n* = 5 mice; *P = 0.0028. Scale bars, 100 µm. **j**, Immunostaining CK19 on liver sections collected at week 7 after NTBC removal. Quantification of ductular reaction in ×10 field is shown on the right panel. Data represent mean ± s.d.; *n* = 5 mice. Scale bars, 100 µm. **k**, Schematic showing inhibition of TLPC-to-hepatocyte following abrogation of WNT signaling. Statistical analysis was performed by two-tailed unpaired Student’s *t* test in **g**–**j**. w, weeks.

scRNA-seq profiling of tdT^+^ BECs, TLPCs and TLPC-derived hepatocytes from *CK19-CreER;Fah-LSL/LSL;R26-tdT* mice at 28 days after NTBC withdrawal showed WNT/β-catenin signaling target genes, such as *Lect2*, *Cyp2e1* and *Cyp1a2*, highly enriched in TLPC-derived hepatocytes as well as in a subset of TLPCs (Fig. 6a–d). Immunostaining confirmed the expression of WNT/β-catenin-regulated CYP2E1 in newly formed hepatocytes from TLPCs at day 28 (Fig. 6e). Considering the higher expression of WNT/β-catenin-regulated genes in TLPC-derived hepatocytes (Fig. 6d,e), *Axin2* expression in BECs from mice with impaired hepatocyte regeneration^44^, and the role of WNT/β-catenin signaling in promoting hepatocyte fate^45^, we hypothesized that WNT/β-catenin signaling may promote transdifferentiation into hepatocytes during liver regeneration. To test this hypothesis, we first generated *CK19-2A-CreER;Fah-LSL/LSL;Ctnnb1*^*fl/fl*^*;R26-Confetti* (mutant) mice, in which TAM induced deletion of β-catenin (resulting in loss of WNT/β-catenin signaling) and the simultaneous expression of a Confetti reporter^31^ in BECs (Fig. 6f). Littermate *CK19-2A-CreER;Fah-LSL/LSL;Ctnnb1*^*fl/+*^*;R26-Confetti* mice were used as controls (Fig. 6f). We collected livers from the mutant and control mice at day 25 and ~7 weeks after NTBC removal for analysis of TLPCs and hepatocytes (Fig. 6f) and confirmed deletion of β-catenin in a subset of BECs (Fig. 6g). Comparable numbers of CK19^+^HNF4α^+^ TLPCs between the mutant and control groups (Fig. 6h) suggest that WNT/β-catenin signaling is not required for BEC-to-TLPC induction. However, it is likely that *Ctnnb1* deletion was not complete in all BECs, which may underestimate the role of WNT/β-catenin signaling in BEC-to-TLPC conversion. The number of BEC-derived hepatocyte clones was significantly reduced in mutant compared with control mice (Fig. 6i). Abundance of CK19^+^ cells remained similar between the mutant and control groups (Fig. 6j), suggesting that WNT/β-catenin signaling is dispensable for a ductular reaction as previously reported^46,47^. These data suggest that WNT/β-catenin signaling is required for efficient hepatocyte transdifferentiation during liver regeneration (Fig. 6k).

**Fig. 7:**
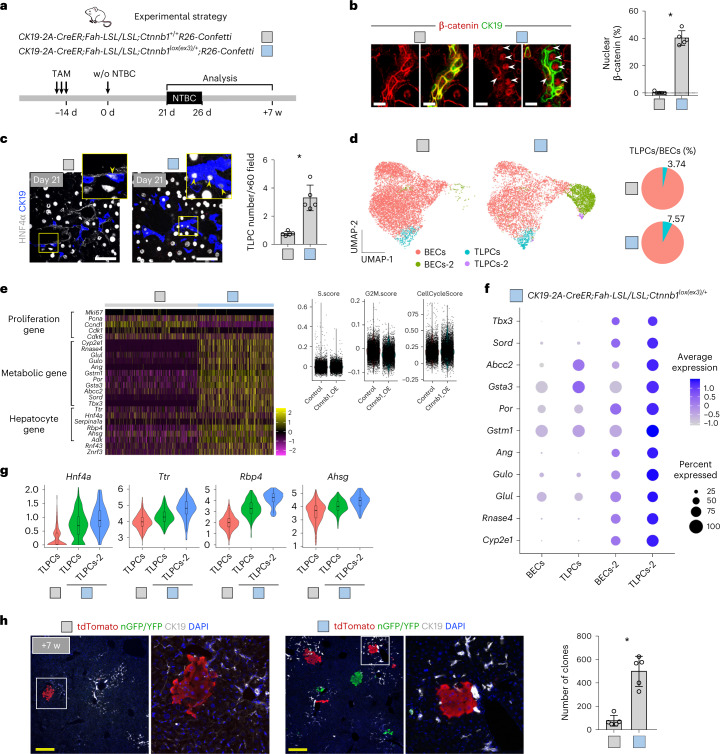
Activation of WNT signaling promotes the activation of TLPCs and their transdifferentiation into hepatocytes. **a**, Schematic showing experimental strategy for tamoxifen, NTBC treatment and tissue collection from C*K19-2A-CreER;Fah-LSL/LSL;Ctnnb1*^*+/+*^*R26-Confetti* mice (*Ctnnb1*^*+/+*^) or *CK19-2A-CreER;Fah-LSL/LSL;Ctnnb1*^*lox(ex3)/+*^*;R26-Confetti* mice (*Ctnnb1*^*lox(ex3)/+*^) at indicated time points. **b**, Immunostaining for β-catenin and CK19 on liver sections. Percentage of nuclear β-catenin is shown on the right panel. Data represent mean ± s.d.; *n* = 5 mice; *P < 0.0001. Scale bars, 10 µm. White arrowheads indicate nuclear β-catenin^+^ BECs. **c**, Immunostaining for HNF4α and CK19 on liver sections collected at day 21 after NTBC removal. Quantification of the number of TLPCs per portal region ×60 field is shown on the right panel. Data represent mean ± s.d.; *n* = 5 mice; *P = 0.0003. Scale bars, 100 µm. Yellow arrowheads indicate TLPCs. **d**, UMAP visualization of the EPCAM^+^ populations collected at day 21 after NTBC removal. The percentage of TLPCs is shown on the right panel. **e**, scRNA-seq heatmap for EPCAM^+^ cells showing expression of selected genes per cell in *Ctnnb1*^*+/+*^ and *Ctnnb1*^*lox(ex3)/+*^ mice. Right panel shows cell cycle score. **f**, Relative expression of metabolic genes in cell clusters identified by scRNA-seq. Circle size represents the within-cluster probability of gene detection and fill colors represent normalized mean expression levels. **g**, Violin plots showing expression levels for selected hepatocyte genes per single cell in TLPC clusters in *Ctnnb1*^*+/+*^ and *Ctnnb1*^*lox(ex3)/+*^ mice. **h**, Immunostaining of tdT, nGFP or YFP and CK19 on liver sections collected at week 7 after NTBC removal. The number of reporter^+^ clones per left liver lobe is shown on the right panel. Data represent mean ± s.d.; *n* = 5 mice; *P = 0.0001. Scale bars, 100 µm. Statistical analysis was performed by two-tailed unpaired Student’s *t* test in **b**, **c** and **h**. w, weeks.

**Extended Data Fig. 10:**
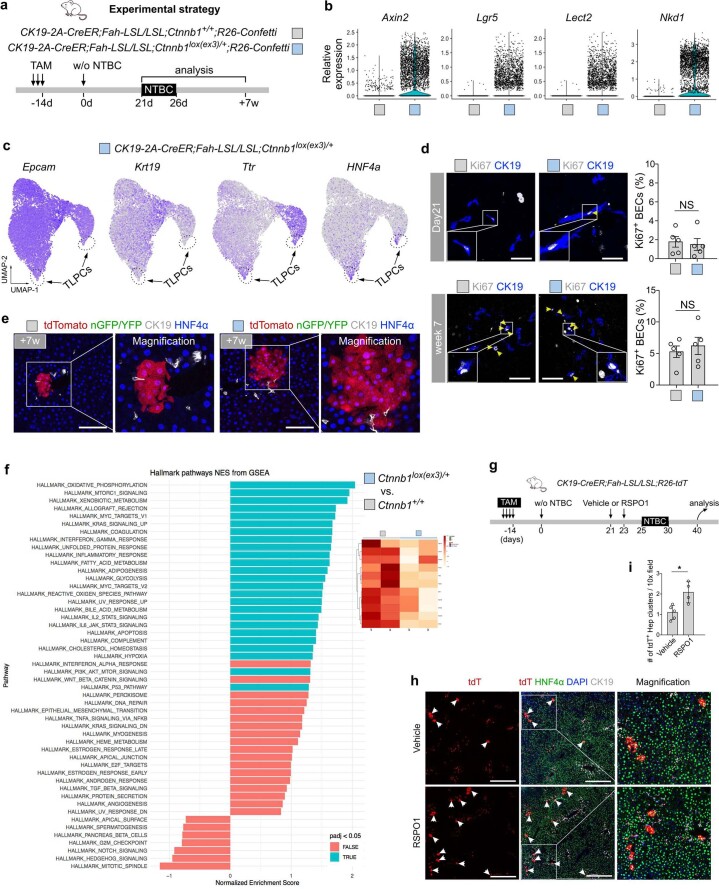
WNT activation increases BEC-to-hepatocyte formation but not BEC proliferation. **a**, Schematic figure showing the experimental strategy for *CK19-2A-CreER;Fah-LSL/LSL;Ctnnb1*^*+/+*^*R26-Confetti* mice (*Ctnnb1*^*+/+*^) or *CK19-2A-CreER;Fah-LSL/LSL;Ctnnb1*^*lox(ex3)/+*^*;R26-Confetti* mice (*Ctnnb1*^*lox(ex3)/+*^). **b**, Violin plots showing selected gene expression levels per single cell in BECs collected from *Ctnnb1*^*+/+*^ and *Ctnnb1*^*lox(ex3)/+*^ mice at day 21. **c**, UMAP plots showing the expression of the indicated genes in each cluster collected from *Ctnnb1*^*lox(ex3)/+*^ mice at day 21. **d**, Immunostaining for CK19 and Ki67 on liver sections collected at day 21 and week 7 from the indicated mice. The percentage of proliferating BECs (Ki67^+^) is shown on the right panel. Data represent mean ± SD; n = 5 mice; NS, no significance. Scale bars, 50 µm. **e**, Immunostaining for fluorescent markers, CK19, and HNF4α on liver sections from the indicated mice. Scale bars, 100 µm. **f**, GSEA analysis in the left panel shows differentially expressed hallmark pathways between the *Ctnnb1*^*lox(ex3)/+*^ group and *Ctnnb1*^*+/+*^ group. Right panel shows heat map of Notch pathway leading edge genes. **g**, Schematic figure showing the experimental strategy. **h**, Immunostaining for tdT, HNF4α, and CK19 on sections of liver collected at day 40. Scale bars, 500 µm. **i**, Quantification of the number of tdT^+^ hepatocyte clusters per 10x field in vehicle or RSPO1-treated mice livers. Arrowheads, tdT^+^HNF4α^+^ hepatocyte clones. Data are the mean ± SD; n = 4 (RSPO1 group) and 5 (vehicle group) mice; *P = 0.0107. Statistical analysis was performed by two-tailed unpaired Student’s *t* test in d, i; Kolmogorov-Smirnov (KS) test was used in f.

We next asked whether activation of WNT/β-catenin signaling promotes hepatocyte transdifferentiation during liver regeneration. Therefore, we generated *CK19-2A-CreER;Fah-LSL/LSL;Ctnnb1*^*lox(ex3)/+*^*;R26-Confetti* (*Ctnnb1*^*lox(ex3)/+*^ group) mice (Fig. 7a), in which TAM-induced deletion of *β-*catenin exon3 resulted in stable (dominant active) β-catenin expression^48^ and the simultaneous expression of the Confetti reporter in BECs. *CK19-2A-CreER;Fah-LSL/LSL;Ctnnb1*^*+/+*^*;R26-Confetti* (*Ctnnb1*^*+/+*^ group) littermates were used as controls. As expected, increased activated (nuclear) β-catenin was detected in *Ctnnb1*^*lox(ex3)/+*^ BECs, whereas *Ctnnb1*^*+/+*^ control BECs showed no activated β-catenin (Fig. 7b). The number of TLPCs significantly increased in *Ctnnb1*^*lox(ex3)/+*^ mice compared with *Ctnnb1*^*+/+*^ mice at day 21 after NTBC removal (Fig. 7c). Next, we performed scRNA-seq on EPCAM^+^ cells isolated from *Ctnnb1*^*lox(ex3)/+*^ and *Ctnnb1*^*+/+*^ mice at day 21 after NTBC removal. WNT/β-catenin target genes (*Axin2*, *Lgr5*, *Lect2* and *Nkd1*) were substantially upregulated in *Ctnnb1*^*lox(ex3)/+*^ BECs (Extended Data Fig. 10a,b). UMAP identified multiple BEC clusters, among which we found a significant increase in TLPCs in the *Ctnnb1*^*lox(ex3)/+*^ group compared with the *Ctnnb1*^*+/+*^ group (7.57% versus 3.74%; Fig. 7d, Extended Data Fig. 10c and Supplementary Table 1). Although WNT/β-catenin signaling is a well-known pro-proliferative signal^49–51^, cell cycle gene signatures were not increased in *Ctnnb1*^*lox(ex3)/+*^ BECs (Fig. 7e). Instead, the expression of metabolic enzymes and other hepatocyte genes was substantially increased in *Ctnnb1*^*lox(ex3)/+*^ compared with *Ctnnb1*^*+/+*^ BECs (Fig. 7e).

In *Ctnnb1*^*lox(ex3)/+*^ cells, UMAP identified a new emerging BEC cluster (BECs-2), as well as a new TLPC cluster (TLPCs-2), both with significant enrichment for the expression of metabolic genes (*Cyp2e1, Rnase4, Glul, Gulo, Gstm1, Gsta3, Por, Abcc2* and *Sord*) (Fig. 7f), which could be due to constitutively active WNT/β-catenin pathway activation. Considering that the expression of hepatocyte genes was upregulated in nonmutant TLPCs compared with BECs following liver injury (Fig. 1c), we next examined hepatocyte gene expression among TLPC clusters in the *Ctnnb1*^*+/+*^ and *Ctnnb1*^*lox(ex3)/+*^ groups. TLPC clusters in *Ctnnb1*^*lox(ex3)/+*^ mice showed higher expression levels of hepatocyte genes than the TLPC cluster in *Ctnnb1*^*+/+*^ mice (Fig. 7g). TLPCs-2 acquired an even higher level of hepatocyte gene expression than TLPCs in the *Ctnnb1*^*lox(ex3)/+*^ group (Fig. 7g). These data indicated that increased WNT/β-catenin signaling activity correlates with the expression of metabolic genes in TLPCs, which likely promote subsequent hepatocyte generation. Proliferation of BECs was unchanged between the *Ctnnb1*^*lox(ex3)/+*^ and *Ctnnb1*^*+/+*^ groups (Extended Data Fig. 10d). At week 7, we observed a significant increase in the number of BEC-derived hepatocyte clones (Fig. 7h and Extended Data Fig. 10e). Additionally, GSEA analysis in isolated BECs revealed a trend for downregulation (yet not significant) of Notch signaling in *Ctnnb1*^*lox(ex3)/+*^ mice compared with *Ctnnb1*^*+/+*^ mice (Extended Data Fig. 10f). Likewise, treatment of *CK19-CreER;Fah-LSL/LSL;R26-tdT* mice with the WNT/β-catenin pathway activator RSPO1 significantly increased the number of BEC-derived hepatocyte clones (Extended Data Fig. 10g–i). Collectively, our data suggest that activation of WNT/β-catenin signaling promotes BEC-to-TLPC transition and transdifferentiation into hepatocytes.

## Discussion

While it is established that hepatocytes can re-enter the cell cycle to proliferate and restore a functional hepatocyte pool in response to various injuries, the contribution of facultative LPCs to this process has been controversial^7,9–11,13,52–55^. The discovery of BEC-to-hepatocyte transdifferentiation in conditions where hepatocyte-mediated regeneration is impaired provided an important new concept enabling liver regeneration^13–18^. Unfortunately, the cellular identity of the BECs with such facultative LPC potential, as well as the molecular mechanisms enabling their transdifferentiation, remained unclear.

We now identified quiescent TLPCs, which are characterized by a hybrid BEC/hepatocyte gene expression signature and represent a transitional LPC state that situates in-between BECs and hepatocytes. Whether all BECs or just a subset of them have the potential to become TLPCs remains unclear. Additional lineage tracing and profiling studies in rats will be necessary to clarify whether oval cells, previously identified in pioneering studies showing hepatocyte transdifferentiation^56–58^, are similar to the TLPCs we identified in mice. It is also possible that additional HNF4α-negative LPCs, identified by other markers, contribute to liver regeneration by restoring hepatocytes. Several other markers have been used to identify putative BEC-associated LPCs, including CD24/CD133 (ref. ^59^), Foxl1 (refs. ^14,54,55^) and Tweak/Fn14 (ref. ^60^). However, validation of TLPCs and uncovering their full differentiation potential requires lineage tracing studies with improved genetic approaches. Using dual genetic lineage tracing to specifically label HNF4α^+^CK19^+^ TLPCs, we now prove that these are a source of newly generated hepatocytes in conditions where hepatocyte regeneration is impaired.

Co-expression of BEC and hepatocyte markers has also been reported in human livers. While classical immunostaining did not identify such mixed populations in healthy adult human livers^61^, tissue-tethered cytometric analyses found CK19^+^ BECs with faint HNF4α expression^62^, possibly representing primed BECs that can upregulate HNF4α to acquire LPC potential when needed. Notably, patients with acute liver failure^41^ or cirrhotic livers in patients with viral hepatitis or AIH^16^ showed strong HNF4α expression in BECs. We now identified HNF4α^+^CK19^+^ BECs in 11 different liver disease indications, whereas we only found negligible amounts in healthy patients. We further found that the induction of these TLPCs correlates with the amounts of senescent hepatocytes and severity of the disease. In cirrhotic livers, generation of hepatocytes by BECs has been proposed to be a major mechanism for parenchymal regeneration^63,64^. Together, this suggests that TLPC induction may be a common mechanism in human liver disease. However, lack of lineage tracing possibilities in patients does not allow for proving their transdifferentiation potential.

**Fig. 8:**
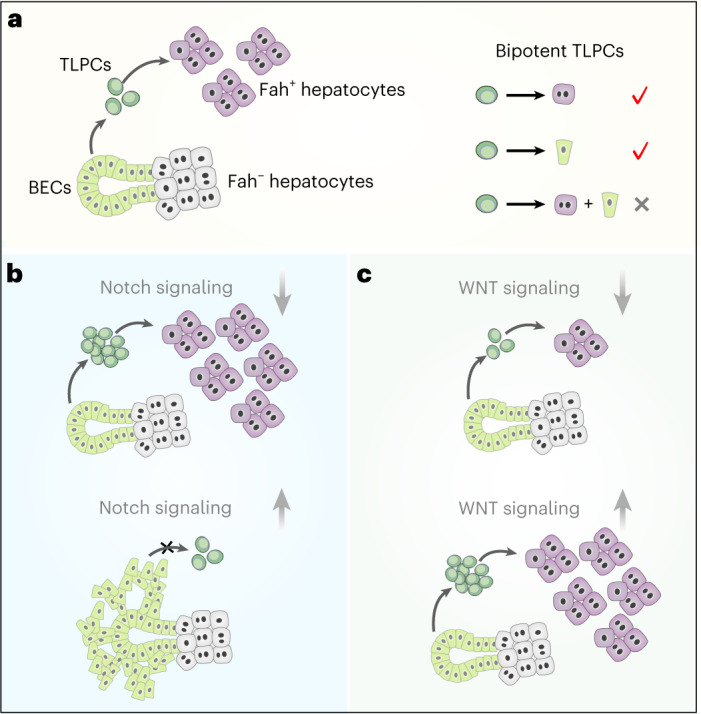
Bipotent TLPCs contribute to liver regeneration. **a**, A cartoon image showing that TLPCs originate from BECs and differentiate into hepatocytes during liver regeneration. TLPCs are bipotent, contributing to either hepatocyte or BEC clones. **b**, Inhibition of Notch signaling promotes BEC-to-TLPC conversion, while activation of Notch signaling inhibits this process while promoting BEC proliferation. **c**, Inhibition of WNT signaling reduces TLPC-to-hepatocyte conversion but does not influence BEC-to-TLPC activation. WNT signaling activation promotes BEC-to-TPLC activation and TLPC-to-hepatocyte conversion.

Mechanistically, we show that WNT/β-catenin and Notch signaling pathways orchestrate the stepwise BEC-TLPC-hepatocyte transdifferentiation process (Fig. 8). Notch signaling is crucial for determining cell lineages in the liver, regulating the differentiation of hepatoblasts to cholangiocytes^38,39,65–70^. We found that inhibition of Notch signaling enhanced BEC-to-TLPC conversion, while increased Notch signaling blocked this process. We further show that activated WNT/β-catenin signaling promoted TLPC-to-hepatocyte conversion and also enhanced the conversion of BECs into TLPCs, consistent with WNT/β-catenin-induced BEC-to-hepatocyte conversion in hepatic organoids^44^, and mice with biliary injury^45^. Conversely, abrogation of WNT/β-catenin signaling blocked TLPC-to-hepatocyte conversion and newly generated hepatocytes express WNT-regulated metabolic enzymes, suggesting that the pathway is key to the transdifferentiation process. Because our genetic tool only allows us to activate WNT/β-catenin signaling in BECs, but not in TLPCs specifically, we could not directly distinguish whether WNT/β-catenin pathway activation affects TLPC-to-hepatocyte or TLPC-to-BEC conversion. Notably, extended survival in injured mice following BEC-to-hepatocyte transdifferentiation, and the potential of the Notch inhibitor DBZ and WNT/β-catenin pathway agonist RSPO1 in enhancing transdifferentiation, suggest possibilities to therapeutically exploit this regenerative mechanism. However, Notch blockade in the liver may impair the biliary system^42^ and RSPO1 treatment impaired metabolic zonation^50^, suggesting a more targeted therapy would be required to benefit patients with liver disease. Together, our data provide the cellular identity and mechanistic cues for transdifferentiation-mediated liver regeneration, establishing a rational basis for potentially therapeutic concepts leveraging this fundamental regenerative process.

## Methods

### Mice

All mice experiments were performed in accordance with the guidelines of the Institutional Animal Care and Use Committee (IACUC) at the Center for Excellence in Molecular Cell Science, Shanghai Institutes of Biological Sciences, Chinese Academy of Science. The approved animal protocol number is SIBCB-S374-1702-001-C1. The *CK19-CreER*, *HNF4α-DreER*, *R26-tdTomato (R26-tdT)*, *R26-RL-tdTomato (R26-RL-tdT)*, *R26-GFP*, *R26-Confetti*, *Ctnnb1*^*fl/+*^, *Ctnnb1*^*lox(e3)/+*^ and *Rbpj*^*fl/+*^ mouse lines were reported previously^30,31,48,71–76^. *Fah-LSL/+*, *CK19-2A-CreER* and *R26-NICD-GFP* mouse lines were generated by homologous recombination using CRISPR–Cas9 as previously described^77^. These new mouse lines were generated by Shanghai Model Organisms Center, Inc. (SMOC). For *Fah-LSL/+*, the cDNA encoding loxp-stop-loxp was inserted into the intron between exon1 and exon2 of the *Fah* gene, followed by polyadenylation sequence. For C*K19-2A-CreER*, the cDNA encoding CreER^T2^ recombinase was inserted into the translation stop codon of the C*K19* gene. A P2A peptide sequence was used to link CK19 coding region and CreER cDNA, ensuring both transcriptions. For *R26-NICD-GFP* mouse line, the CAG-loxp-stop-loxp-NICD-2A-GFP cDNA was generated and inserted into Rosa26 locus as *R26-NICD-GFP*. All mice involved in the study were maintained on 129/C57BL6 and ICR mixed background. All *Fah-LSL/LSL* mice were kept with 7.5 μg ml^−1^ NTBC (2-(2-nitro-4-trifluoro-methylbenzoyl)-1,3-cyclohexanedione) dissolved in drinking water. Adult mice received 0.2 mg TAM per gram mouse body weight by oral gavage for indicated times and at indicated times. Of note, *CK19-2A-CreER;R26-NICD-GFP* mice and *CK19-2A-CreER;R26-tdT* mice used as control were treated with 0.05 mg TAM per gram mouse body weight; *CK19-CreER;HNF4α-DreER;Fah-LSL/LSL;R26-RL-tdT*, *CK19-CreER;Fah-LSL/LSL;R26-RL-tdT* and *HNF4α-DreER;Fah-LSL/LSL;R26-RL-tdT* mice were treated with 0.1 mg TAM per gram mouse body weight. To avoid mice dying from intestinal infections, we supplied *CK19-2A-CreER;Fah-LSL/LSL;Ctnnb1*^*fl/+*^ and *CK19-2A-CreER;Fah-LSL/LSL;Ctnnb1*^*fl/fl*^ mice with 0.005% enrofloxacin in drinking water during experiments. Mice, both males and females, at the age of 8–20 weeks were used for experiments with similar-aged mice for both control and experimental groups. All mice were housed at the laboratory Animal center of the Center for Excellence in Molecular Cell Science in a Specific Pathogen Free (SPF) facility with individually ventilated cages. The room has controlled temperature (20–25 °C), humidity (30–70%) and light (12 hours light-dark cycle). For mouse survival study (Extended Data Figs. 1g and 3b), we did not analyze mice after they died in these experiments. No data in mice experiments were excluded.

### Genomic PCR

Genomic DNA was extracted from the mouse tail. Tissues were lysed with Proteinase K overnight at 55 °C, followed by centrifugation at maximum speed for 8 min. DNA was deposited with isopropanol and washed in 70% ethanol. All mice were genotyped with specific PCR primers that distinguish knock-in alleles from wild-type alleles. Sequences of all primers were included in Supplementary Table 5.

### Immunostaining

Immunostaining was performed as previously described^78^. In detail, livers were fixed in 4% PFA at 4 °C for 1 h, then washed in PBS and dehydrated in 30% sucrose overnight at 4 °C and embedded in OCT (Sakura). For staining, the cryosections were washed in PBS, incubated in blocking buffer (5% normal donkey serum (Jackson Immunoresearch), 0.1% Triton X-100 in PBS) for 30 min at room temperature then stained with the primary antibodies overnight at 4 °C. Signals were developed with Alexa fluorescence antibodies (Invitrogen). HRP-conjugated antibodies with tyramide signal amplification kit (PerkinElmer) were used to amplify weak signals. HRP-conjugated antibodies with ImmPACT DAB kit (Vector lab, SK-4105) were used to show CK19 density. Nuclei were counterstained with 4'6-diamidino-2-phenylindole (DAPI, Vector lab). The following antibodies were used: tdT (tdTomato, Rockland, 600-401-379, 1:500; or Rockland, 200-101-379, 1:500), GFP (Invitrogen, A11122; 1:500), GFP (Rockland, 600-101-215M; 1:500), GFP (nacalai tesque, 04404-84; 1:500), p21 (Abcam, ab188224; 1:500), Ki67 (Abcam, ab15580; 1:200), CK19 (Developmental Studies Hybridoma Bank, TROMA-III, 1:500), CK19 (Abbomax, 602-670; 1:500), HNF4α (Cell Signalling, 3113s; 1:500), HNF4α (Abcam, ab41898; 1:100), FAH (Abclonal, A13492; 1:500), *β*-catenin (BD Pharmingenp; 1:100), anti-active-*β*-catenin (Millipore, Upstate, 05-665; 1:100), GS (Abcam, Ab49873; 1:1,000), E-cadherin (E-cad, Cell signaling, 3195; 1:100), CYP2E1 (Abcam, ab28146; 1:100), Sox9 (Millipore, AB5535; 1:1,000), YAP/TAZ (Cell signaling, 8418; 1:100), Mucin2 (Santa Cruz, sc-15334; 1:400), Epcam (Abcam, ab92382; 1:400), OPN (R&D, AF808-SP; 1:500), A6 (a gift from Valentina Factor; 1:100). CK19 density was calculated based on measuring the percentage of CK19 area in each field. Immunostaining images were acquired by Zeiss confocal microscope (LSM710) or Nikon A1 confocal microscope. For quantification of the hepatocytes clone number per left lobe, serial liver sections (20 μm) were used for staining. For TLPC number quantification, we collected ten tissue sections (20 tissue sections in *Ctnnb1* gene knockout experiment) from each mouse liver and took five random fields from each liver section for quantification. Immunostainings for CK19 and HNF4α were performed on both mutant and control liver sections at the same time to avoid potential batch differences during staining. Imaging of all immunostained slides was performed under the same exposure and contrast conditions using the same confocal microscope.

### DBZ and RSPO1 treatment

To study the effect of dibenzazepine (DBZ; MedChemExpress, HY-13526) that inhibits Notch cleavage and blocks activated Notch signaling on the conversion of BECs to hepatocytes, *CK19-CreER;Fah-LSL/LSL;R26-tdT* mice were treated with tamoxifen for four times at indicated time and then treated with either DBZ (0.01 mg g^−1^) or oil (control) by oral gavage according to the schematic figure shown in the Extended Data Fig. 8a. To study the effect of RSPO1 (MedChemExpress, HY-13526 or Peprotech, 120-38) induced WNT/β-catenin activation on the conversion of BECs to hepatocytes, *CK19-CreER;Fah-LSL/LSL;R26-tdT* mice were treated with tamoxifen for four times at indicated time and then injected intraperitoneally with either 20 mg kg^−1^ RSPO1 or PBS (control) according to the schematic figure shown in the Extended Data Fig. 10g.

#### H&E staining

Cryosections were washed in PBS for 5 min to remove OCT, then incubated in hematoxylin A for 10 min, followed by washing in water. Then, cryosections were incubated in 1% concentrated hydrochloric acid diluted in 70% ethanol for 1 min and washed in water. Afterward, the sections were incubated in 1% ammonia water for 1 min, followed by washing in water. The sections were stained with Eosin-Y solution for 10 s and dehydrated in ethanol and xylene. Finally, sections were mounted with resinous medium. Images were acquired using an Olympus microscope (Olympus, BX53).

#### Cell isolation and fluorescence-activated cell sorting

Liver cells were isolated by standard two-step collagenase perfusion as described previously^33^. Briefly, mice were anesthetized and the liver was exposed through an incision in the lower abdomen. A needle was inserted into the inferior vena cava and secured with a hemostatic clamp around the needle. Portal vein was cut immediately when the mouse liver was perfused with perfusion medium using a peristaltic pump. Then, the liver was next perfused with medium containing collagenase type I (150 U ml^−1^; Invitrogen) for 10 min to adequately digest the liver. After removing the gallbladder, the liver was dissected with cold resuspension buffer (0.5% BSA and 2 mM EDTA in PBS) to free the hepatic cells. Then the cell suspension was passed through a 70-μm cell strainer (BD Biosciences, 352350) and centrifuged at 50*g* for 3 min at 4 °C. The non-parenchymal cells that remained in supernatant were collected and passed through a 40 μm cell strainer (BD Biosciences, 352340), then centrifuged at 400*g* for 5 min at 4 °C. The cell pellet was resuspended in red blood cell lysis buffer (eBioscience, 00-4333-57) for 5 min at room temperature and washed with cold resuspension buffer and centrifuged at 400*g* for 5 min at 4 °C. The washing step was repeated once again. Subsequently, cells were stained with the positive selection antibody (anti-mouse EPCAM-APC; eBioscience, 17-5791-82) diluted in resuspension buffer for 30 min in the dark at 4 °C. After staining, cells were washed with resuspension buffer and centrifuged at 400*g* for 5 min. EPCAM^+^ cells were enriched by using APC microbeads (130-090-855, Miltnyi Biotec) according to the manufacturer’s protocols before sorting with Sony MA900 equipped with a 100 μm nozzle in purity mode. Cell viability was assessed with DAPI staining. EPCAM^+^ cells were isolated for further scRNA-seq of BECs or bulk RNA-seq or qRT-PCR. For scRNA-seq of tdT^+^ cells (Fig. 6a–c), cells were not stained with anti-mouse EPCAM-APC antibody. Cells were sorted based on the expression of tdT.

### Human samples and IHC analysis

Glass slides with formalin-fixed and paraffin-embedded (FFPE) sections from patient livers (healthy *n* = 6, ASH cirrhosis *n* = 5, acute liver failure *n* = 9, nonalcoholic steatohepatitis (NASH) noncirrhosis *n* = 6, NASH cirrhosis *n* = 6, hepatitis B (HepB) noncirrhosis *n* = 6, HepB cirrhosis *n* = 6, Hepatitis C (HepC) noncirrhosis *n* = 6, HepC cirrhosis *n* = 5, AIH *n* = 5, PSC *n* = 5 and PBC *n* = 5) were obtained from the University Hospital Basel Tissue Bank. Healthy livers were classified by normal morphology during histopathological assessment. The biopsies were originally acquired for routine diagnostic and patients signed a general informed consent for the use of remaining tissue for research purposes in accordance with the Swiss Federal Human Research Act (HRA). Patients did not receive compensation. The study was approved by the ethics committee of Northwest and Central Switzerland (EKNZ) as part of the EKNZ. FFPE blocks were cut in 3-μm thickness and sections were placed on silanized/charged slides. Slides were dried for 15-20 min at 37 °C. Immunohistochemistry for p21 was performed using a Ventana Benchmark XT (Ventana). Antigen retrieval was performed with CC1 (Ventana, 950–500) for 16 min, prediluted primary antibody for anti-p21 (Ventana, 760-4453; mouse monoclonal) was incubated for 24 min then visualized with the OptiView-DAB detection kit (Ventana). HNF4α/CK19 costaining was conducted on a Ventana Discovery Ultra (Roche Diagnostics). Antigen retrieval was performed with CC1 (Ventana, 950–500) for 40 min. Anti-HNF4α (Cell Signaling Technology, 3113S; rabbit monoclonal) was incubated for 56 min, detected with OmniMap anti-Rb HRP (Ventana, 760-4311) and visualized with Discovery Purple HRP Kit (Ventana, 760-229). Subsequently, anit-CK19 (Ventana, 760-4281; mouse monoclonal) was applied for 52 min, detected with UltraMap anti-Ms Alk Phos (Ventana, 760-4312) and visualized with Discovery Yellow AP Kit (Ventana, 760-239). Stained slides were scanned with an Aperio ScanScopeXT and visualized using Aperio Image Scope software (Leica Biosystems). Two samples were excluded from analyses due to staining quality challenges (1 ALF patient sample for p21 assessment and 1 patient sample for CK19/HNF4α assessment). The percentage of CK19^+^ cells expressing HNF4α, as well as the percentage of hepatocytes with nuclear p21 staining, were counted manually, respectively. Statistical analyses were performed using GraphPad Prism software.

### scRNA-seq and bioinformatics analysis

#### scRNA-seq

Isolated cell suspension was loaded to the 10X Chromium and ~8,000 cells were expected to be captured when Gel Beads-in-emulsions were generated. The library was prepared followed by the instruction manual of Single Cell 3′ Gene Expression kit (v3.1) or Single Cell 5′ Gene Expression kit (v2). Briefly, the Gel Beads-in-emulsions were first incubated and reverse transcripted to first-strand cDNA. The single-strand cDNA was purified by Dynabeads and amplified using 12 cycles to generate the double strands cDNA. Next, dsDNA was fragmented, end-repaired and further ligated with adaptor. Lastly, index PCR was performed before sequencing. The library was sequenced on the Illumina Hiseq X ten PE150 platform.

#### Single-cell transcriptomic analysis

Sequencing reads were aligned, annotated and demultiplexed using CellRanger (v4.0.0) with the mm10-2020-A reference provided by 10X Genomics. Further downstream analyses were carried out using the Seurat R package (v4.0.5)^79^. Quality control was performed using the subset function using the threshold of nFeature_RNA larger than 2,000 and less than 8,000, nCount_RNA larger than 8,000 and less than 50,000, as well as percentage of expressed mitochondrial gene less than 10% to filter out low-quality cells and potential doublets. PCA was calculated using the scaled expression data of 3,000 most variable genes, which were selected by ‘vst’ method using FindVariableFeatures function. Dimension reduction and clustering were further performed. Different dims of PCA and different values of resolution parameters were tested^80^. We set the final resolution to 0.2 (testing a range from 0.1 to 0.5) and dims to 15 (testing a range from 10 to 20) first in the sample of *CK19-CreER;Fah-LSL/LSL* mice. Given that the obtained clustering sensitivity for a given resolution is dependent on the number of cells of that subpopulation in each respective sample, we swept over the same range of resolutions for the other samples to assure the proportion of TLPCs is comparable with the statistics result.

#### DEGs and pathway enrichment

Two-sided Wilcoxon rank-sum test was used to define marker genes for clusters and samples using the FindMarkers function in Seurat and *P* values were Benjamin-Hochberg FDR correction for the total number of comparisons. The GO BP pathway enrichment analyses of DEGs calculated above are performed using Metascape webtool^81^.

#### Data integration

To compare the scRNA-seq data from the sample of *CK19-CreER;Fah-LSL/LSL* mice and two published samples of mice with DDC-induced injury, data integration was performed using the MNN algorithm^82^. In detail, QC filter and preprocessing were performed as described in the original articles^32,33^. The *RunFastMNN* function in SeuratWrappers package was used to integrate these three datasets. The highest 2,000 variable features were selected to correct the batch effects between samples. The dimensions of the first 19 MNNs and resolution of 0.22 were used to unsupervised cluster all cells. The first 19 MNNs were used to reduce dimensions by *RunUMAP* function. Other datasets were also integrated by the *RunFastMNN* function. The same number of MNN dimensions but different resolutions were used for clustering and dimension reduction.

#### Trajectory

To map the differentiation trajectory directions, scVelo was used to calculate the RNA velocity^83^. In brief, the cell filter mentioned above was used to re-UMAP using the spliced assay data. Genes less than 20 counts were filtered out and 1,500 highly variable genes were retained and log-normalized. 30 PCs and 30 neighbors were used to compute moments based on connectivity, and then calculate velocities for each individual cell. The velocity embedding stream plot was drawn and colored by Seurat clusters.

#### Cell cycle scoring for scRNA-seq

To evaluate the potential ability of proliferation for each cell, we first calculate the S.score and G2M.score for each single-cell data using the CellCycleScoring function in Seurat package. Also, we used enrichIt function in escape^84^ package to perform ssGSEA analysis to calculate the cell cycle score using the union gene set of S phage gene set and G2M phage gene set. These scores of BECs were all upregulated in *CK19-2A-CreER*;*Fah-LSL/LSL;R26-NICD-GFP* mice compared to *CK19-2A-CreER;Fah-LSL/LSL;R26-tdT* mice. The changes in these scores were inconsistent in the comparison of *CK19-2A-CreER;Fah-LSL/LSL;Ctnnb1*^*lox(ex3)/+*^ mice and *CK19-2A-CreER;Fah-LSL/LSL;Ctnnb1*^*+/+*^ mice. G2M.score was slightly higher in BECs of *CK19-2A-CreER;Fah-LSL/LSL;Ctnnb1*^*+/+*^ mice, while cell cycle score was slightly higher in *CK19-2A-CreER;Fah-LSL/LSL;Ctnnb1*^*lox(ex3)/+*^ mice. All gene sets used in this paper are listed in Supplementary Table 4.

#### Bulk RNA-seq and data analysis

Total RNA was extracted from BECs isolated from TAM-treated *CK19-2A-CreER;Fah-LSL/LSL;Ctnnb1*^*+/+*^ and *CK19-2A-CreER;Fah-LSL/LSL;Ctnnb1*^*lox(e3)/+*^ mice at day 21 after NTBC removal. The cDNA library samples were sequenced by BGISEQ platform using PE150. The fastq files were then trimmed by Trim Galore with parameter ‘-q 20 --phred33 --stringency 3 --length 20 -e 0.1’. The trimmed fastq files were further mapped to mouse reference genome GRCm38 (mm10) using STAR^85^ with parameter ‘--outStd SAM --outSAMattributes NH HI AS nM MD --outSJfilterReads Unique --runThreadN 12 --outFilterMismatchNoverLmax 0.04 --outFilterMismatchNmax 999 --sjdbOverhang 149’. The generated SAM files were converted and sorted to BAM files by samtools, which were calculated by featureCounts from the Subread package^86^ and generated count matrix for each gene. The count matrix was input to DESeq2 (V1.6.3)^87^ for differential gene expression analysis, based on a model using negative binomial distribution. The DEseq2 result was taken to assess the enrichment of hallmark pathways using fgsea package for preranked GSEA.

#### GSEA analysis

The GSEA analysis of hallmark pathways between *CK19-2A-CreER;Fah-LSL/LSL;Ctnnb1*^*+/+*^ and *CK19-2A-CreER;Fah-LSL/LSL;Ctnnb1*^*lox(e3)/+*^ mice was done by fgesa package, which used the Kolmogorov-Smirnov (KS) test to evaluate the enrichment of a gene set in a ranked list of genes. To perform the KS test, the fgsea package first ranks the genes in the gene set and the genes outside of the gene set by their statistical significance (for example, *P* values). The cumulative distribution functions of the ranked genes in the gene set and the ranked genes outside of the gene set are then calculated. The maximum difference between these two cumulative distribution functions is then calculated and used as the test statistic.

#### RNA isolation and quantitative RT-PCR

Total RNA was extracted from the liver of indicated mice or BECs isolated from indicated mice treated with TAM or oil. Cells were lysed with Trizol (Invitrogen, 15596018), and total RNA was extracted according to the manufacturer’s instructions. Then, RNA was reverse-transcribed into cDNA using Prime Script RT kit (Takara, RR047A). The SYBR Green qPCR master mix (Thermo Fisher Scientific, 4367659) was used and quantitative RT-PCR was performed on QuantStudio 6 Real-Time PCR System (Thermo Fisher Scientific). *Gapdh* was used as internal control. For qPCR of *Fah* gene, the forward primer for qPCR is located in exon7 and the reverse primer is located in exon8, and their PCR produced is 74 bp overlapping part of exon7 and exon8. Sequences of all primers are included in Supplementary Table 5.

#### Western blot

Liver tissues were collected at the indicated stages. All samples were lysed in RIPA lysis buffer (Beyotime, P0013B) containing protease inhibitors (Roche, 11836153001) for 30 min on ice, and then centrifuged at 13,500*g* for 5 min to collect the supernatant. All samples were mixed with loading buffer (Beyotime, p0015L) and boiled for 10 min. Because the molecular weights between FAH (46 kDa) and GAPDH (36 kDa) are close, we loaded the same amount of protein into two gels to detect FAH and GAPDG, respectively. Western blot analyses were performed with precast gradient gels (Beyotime, P0469M) and transferred onto polyvinyli denefluoride membranes (Millipore, IPVH00010). After blocking in PBST containing 5% BSA, the membranes were incubated with primary antibodies overnight at 4 °C, then washed three times and incubated with HRP-conjugated secondary antibodies. Signals were detected by incubating with chemiluminescent HRP substrate (Thermo Fisher Scientific, WBKLS0500). The following antibodies were used: FAH (Abclonal, A13492; 1:500), GAPDH (Proteintech, 60004-1-IG; 1:2,000), Peroxidase AffiniPure Goat Anti-Rabbit IgG (Jackson ImmunoResearch, 111-035-047; 1:4,000) and Peroxidase AffiniPure Donkey Anti-Mouse IgG (Jackson ImmunoResearch, 715-035-150; 1:4,000).

#### Serum biochemical analysis

The blood was collected from indicated mice and centrifuged at 850*g* for 15 min at 4 °C. The serum that remained in the supernatant was collected for biochemical analyses. ALT and AST were measured by 7600 clinical analyzer (Hitachi) or 4600 fully automatic biochemical analyzer (VITROS). TBIL was measured by 4600 fully automatic biochemical analyzer (VITROS).

#### Statistics

For image acquisition, as well as analyses such as quantification by IF and IHC of cell number or CK19 density, the investigators were blinded. Investigators were not blinded to mouse treatment and sacrifice because mouse treatment and sacrifice were performed by the same people. Investigators were not blinded for scRNA-seq analysis studies as there were no separate groups and the samples were annotated. For western and qPCR, the investigators were not blinded to the loading samples. Within an experimental condition, the allocation of mice was random. Data were presented as means ± s.d. Statistical analysis was performed by two-tailed unpaired Student’s *t* test for comparison of differences between two groups, and by ANOVA followed by Tukey’s method for multiple comparisons. *P* < 0.05 was considered to be statistically significant. The *P* value was added in the figure legend for each comparison, with statistical method included. Each image in Fig. 1e is representative of five individual mice samples. Each image in Fig. 1f is representative of five individual mice samples. Each image in Fig. 2c is representative of five individual mice samples. Each image in Fig. 2d is representative of five individual mice samples. Each image in Fig. 3e is representative of five individual mice samples. Each image in Fig. 3f is representative of five individual mice samples. Each image in Fig. 3g is representative of five individual mice samples. Each image in Fig. 3h is representative of five individual mice samples. Each image in Fig. 3i is representative of five individual mice samples. Each image in Fig. 4e is representative of five individual mice samples. Each image in Fig. 4f is representative of five individual mice samples. Each image in Extended data Fig. 1i is representative of five individual mice samples. Each image in Extended Data Fig. 1j is representative of five individual mice samples. Each image in Extended Data Fig. 3c is representative of five individual mice samples. Each image in Extended Data Fig. 3e is representative of five individual mice samples. Each image in Extended Data Fig. 3f is representative of five individual mice samples. Each image in Extended Data Fig. 6d is representative of four individual human samples. Each image in Extended Data Fig. 8b is representative of five individual mice samples. Each image in Extended Data Fig. 8c is representative of five individual mice samples. Each image in Extended Data Fig. 9d is representative of five individual mice samples. Each image in Extended Data Fig. 9e is representative of five individual mice samples. Each image in Extended Data Fig. 10e is representative of five individual mice samples.

### Reporting summary

Further information on research design is available in the Nature Portfolio Reporting Summary linked to this article.

## Online content

Any methods, additional references, Nature Portfolio reporting summaries, source data, extended data, supplementary information, acknowledgements, peer review information; details of author contributions and competing interests; and statements of data and code availability are available at 10.1038/s41588-023-01335-9.

## Supplementary information

Reporting Summary

Supplementary Tables Supplementary Tables 1–5.

## Data Availability

All of the data generated or analyzed during this study are included in Figs. 1–8, Extended Data Figs. 1–10 and Supplementary Tables 1–5. scRNA-seq data that support this study have been deposited in the Gene Expression Omnibus (GEO; BioProject ID: PRJNA812361). Bulk RNA-seq data have been deposited in the GEO (NCBI BioProject ID: PRJNA871936). Two published DDC-induced liver injury datasets used in this paper are accessible under accession number GEO: GSE125688 (ref. ^32^) and SRA: PRJNA384008 (ref. ^33^). Source data are provided with this paper.

## Source data

Source Data Fig. 1 Unprocessed western blots for Extended Data Fig. 1d.

## Code availability

This study did not generate any unique code or algorithm. The algorithms used for the analysis during this study are all publicly available. The code of single cell data processing and analysis in this study have been deposited in Zenodo (10.5281/zenodo.7576366).
