# Author Correction: Bipotent transitional liver progenitor cells contribute to liver regeneration

**DOI:** 10.1038/s41588-023-01393-z

**Published:** 2023-04-18

**Authors:** Wenjuan Pu, Huan Zhu, Mingjun Zhang, Monika Pikiolek, Caner Ercan, Jie Li, Xiuzhen Huang, Ximeng Han, Zhenqian Zhang, Zan Lv, Yan Li, Kuo Liu, Lingjuan He, Xiuxiu Liu, Markus H. Heim, Luigi M. Terracciano, Jan S. Tchorz, Bin Zhou

**Affiliations:** 1 State Key Laboratory of Cell Biology, Shanghai Institute of Biochemistry and Cell Biology, Center for Excellence in Molecular Cell Science, Chinese Academy of Sciences, University of Chinese Academy of Sciences, Shanghai, China; 2 Novartis Institutes for BioMedical Research, Novartis Pharma AG, Basel, Switzerland; 3 Institute of Medical Genetics and Pathology, University Hospital Basel, Basel, Switzerland; 4 Key Laboratory of Systems Health Science of Zhejiang Province, School of Life Science, Hangzhou Institute for Advanced Study, University of Chinese Academy of Sciences, Hangzhou, China; 5 School of Life Sciences, Westlake University, Hangzhou, China; 6 Department of Biomedicine, University Hospital and University of Basel, Basel, Switzerland; 7 Clarunis University Center for Gastrointestinal and Liver Diseases, Basel, Switzerland; 8 Department of Biomedical Sciences, Humanitas University, Milan, Italy; 9 IRCCS Humanitas Research Hospital, Milan, Italy; 10 School of Life Science and Technology, ShanghaiTech University, Shanghai, China; 11 New Cornerstone Science Laboratory, Shenzhen, China

**Keywords:** Stem cells, Stem-cell research, Liver diseases

Correction to: *Nature Genetics* 10.1038/s41588-023-01335-9. Published online 13 March 2023.

In the version of this article originally published, reference errors that arose during revisions have now been corrected as follows. In the first sentence of the Results subsection “Bipotent TLPCs differentiate into hepatocytes or BECs,” Thakur, 2019 (*Hepatology* **70**, 1360–1376 (2019)) was cited in error and has now been removed. In the third sentence of the Results subsection “WNT/β-catenin signaling promotes TLPC-to-hepatocyte conversion,” now reading in part “Considering the higher expression of WNT/β-catenin-regulated genes … and the role of WNT/β-catenin signaling in promoting hepatocyte fate,” a citation after “hepatocyte fate” has been changed from Sekiya, 2011 (*Nature* **475**, 390–393 (2011)) to Boulter, 2012 (*Nat. Med*. **18**, 572–579 (2012)). In the last paragraph of the Discussion, the second sentence now includes three new citations (Minnis-Lyons, *Sci. Signal*. **14**, eaay9185 (2021); Cordero-Espinoza, *Cell Stem Cell* **28**, 1907–1921 (2021); and Jörs, *J. Clin. Invest*. **125**, 2445–2457 (2015)), and the end of the fourth sentence has been amended to include a citation to Boulter, 2012. The changes are reflected in the HTML and PDF versions of the article.

