# Supplementary material for: Bipotent transitional liver progenitor cells contribute to liver regeneration
Source: Nat Genet. 2023 Mar 13;55(4):651–64. doi: 10.1038/s41588-023-01335-9 (PMC10101857; doi:10.1038/s41588-023-01335-9)
Supplement: Supplementary file 1 — Reporting Summary [file 41588_2023_1335_MOESM1_ESM.pdf]

nature portfolio

Corresponding author(s): Jan Tchorz, Bin Zhou

Last updated by author(s): Dec 8, 2022

Reporting Summary

Nature Portfolio wishes to improve the reproducibility of the work that we publish. This form provides structure for consistency and transparency in reporting. For further information on Nature Portfolio policies, see our Editorial Policies and the Editorial Policy Checklist.

Statistics

For all statistical analyses, confirm that the following items are present in the figure legend, table legend, main text, or Methods section.

n/a	Confirmed	
☐	☒	The exact sample size (*n*) for each experimental group/condition, given as a discrete number and unit of measurement
☐	☒	A statement on whether measurements were taken from distinct samples or whether the same sample was measured repeatedly
☐	☒	The statistical test(s) used AND whether they are one- or two-sided<br>*Only common tests should be described solely by name; describe more complex techniques in the Methods section.*
☒	☐	A description of all covariates tested
☒	☐	A description of any assumptions or corrections, such as tests of normality and adjustment for multiple comparisons
☐	☒	A full description of the statistical parameters including central tendency (e.g. means) or other basic estimates (e.g. regression coefficient) AND variation (e.g. standard deviation) or associated estimates of uncertainty (e.g. confidence intervals)
☐	☒	For null hypothesis testing, the test statistic (e.g. *F*, *t*, *r*) with confidence intervals, effect sizes, degrees of freedom and *P* value noted<br>*Give P values as exact values whenever suitable.*
☒	☐	For Bayesian analysis, information on the choice of priors and Markov chain Monte Carlo settings
☒	☐	For hierarchical and complex designs, identification of the appropriate level for tests and full reporting of outcomes
☒	☐	Estimates of effect sizes (e.g. Cohen's *d*, Pearson's *r*), indicating how they were calculated

Our web collection on statistics for biologists contains articles on many of the points above.

Software and code

Policy information about availability of computer code

Data collection	Zeiss stereoscope (Axio Zoom. V16) were used for whole-mount bright-field and fluorescence image collection. Olympus fluorescence microscope (B53) were used for H&E picture collection. Zeiss LSM880 confocal and Nikon A1 confocal were used for immunofluorescence data collection. Sony MA900 Flow Cytometer were used for FACS data collection. QuantStudio 6 Real-Time PCR System (Thermo Fisher Scientific) was used for quantitative RT-PCR data collection. Aperio AT2 scanner version 102.0.7.5 and Aperio Image Scope software v12.4.0.5043 (Leica Biosystems) were used for immunostaining data of human tissue samples.
Data analysis	Image J (2.0.0-rc-30/1.49t) and Photoline (18.5.1) were used for immunofluorescence and bright-filed images analysis. FlowJo (X 10.07r2) were used for FACS data analysis. GraphPad Prism 6.2 was used for data analysis. Cellranger (v4.0.0), Seurat R package (v4.3.0), SeuratWrappers(v0.3.0), scVelo (v0.2.3), escape R package (v1.8.0), Metascape were used for scRNA-seq analysis. Trim Galore (v0.6.7), STAR (v2.7.9a), samtools (v1.13), Subread (v2.0.1), DEseq2 R package (v1.32.0), fgsea R package (v1.18.0) were used for bulk RNA-seq analysis.

For manuscripts utilizing custom algorithms or software that are central to the research but not yet described in published literature, software must be made available to editors and reviewers. We strongly encourage code deposition in a community repository (e.g. GitHub). See the Nature Portfolio guidelines for submitting code & software for further information.

Data

Policy information about availability of data

All manuscripts must include a data availability statement. This statement should provide the following information, where applicable:

- Accession codes, unique identifiers, or web links for publicly available datasets
- A description of any restrictions on data availability
- For clinical datasets or third party data, please ensure that the statement adheres to our policy

All data generated by this study are included in this article and it's supplementary materials. Source data are provided with this paper. scRNA-seq data that support this study have been deposited in the Gene Expression Omnibus (GEO) (BioProject ID: PRJNA812361). Bulk RNA-seq data have been deposited in the Gene Expression Omnibus (GEO) (NCBI BioProject ID: PRJNA871936). Two published DDC-induced liver injury dataset used in this paper are accessible under accession number GEO: GSE125688 and SRA: PRJNA384008.

Field-specific reporting

Please select the one below that is the best fit for your research. If you are not sure, read the appropriate sections before making your selection.

☒ Life sciences ☐ Behavioural & social sciences ☐ Ecological, evolutionary & environmental sciences

For a reference copy of the document with all sections, see nature.com/documents/nr-reporting-summary-flat.pdf

Life sciences study design

All studies must disclose on these points even when the disclosure is negative.

Sample size	All experiments were repeated at least three times with similar results, except for single cell RNAseq. Each sample size were described in detail in each figure legend. No statistical methods were used to predetermine the sample size. A minimun number of animals were used according to standard scientific conventions. For sample of scRNA-seq, we reported 7853 single cells (Figure 1b), 11301 single cells in Control group and 6315 single cells in NICE-OE group (Figure 5e), 6140 single cells (Figure 6c),7492 single cells in Ctnnb1 +/+ group and 6326 single cells in Ctnnb1 lox(ex3)/+ group (Figure 7d), 4076 single cells in w/ NTBC group and 7853 single cells in w/o NTBC group (Extended Data Fig 3d). The final single cell numbers were used for scRNA-seq analyses corresponded to each sample size after discarding cells due to bad quality or contanminant population during sorting.
Data exclusions	Two human samples were excluded from analyses due to staining quality challenges (1 ALF patient sample for p21 assessment, 1 patient sample for CK19/HNF4α assessment). For mice survival study (Extended Data Fig. 1g,3b), we did not analyze mice after they died in these experiments. No data in mice experiments was excluded.
Replication	For each animal experiment, at least 3 repeats were done to confirm the reproducibility of the findings. Stainings on patient samples were performed once but at least on 5 patients liver biopsies (each on individual glass slides) per disease indication. n means biological replicates (number of mice or patients) and is indicated in the manuscript. Replicated experiments yielded reproducible results.
Randomization	For all animal experiments, experimental and control animals were randomly allocated from the appropriated genotype. Sample were allocated randomly to different experimental groups. Patient biopsies were selected by the pathologists based on availability and assigned to the different groups by disease pathology without considering covariates.
Blinding	For image acquisition as well as analyses such as quantificaiton by IF and IHC of cell number or CK19 density, the investigators were blinded. Investigators were not blinded to mouse treatment and sacrifice because mouse treatment and sacrifice were performed by same people. Investigators were not blinded for single cell RNA-seq analyses studies as there were not separate groups or the sample were annotated. For western and qPCR, the investigators were not blinding when loading the sample to display the results in a logical way.

Reporting for specific materials, systems and methods

We require information from authors about some types of materials, experimental systems and methods used in many studies. Here, indicate whether each material, system or method listed is relevant to your study. If you are not sure if a list item applies to your research, read the appropriate section before selecting a response.

Materials & experimental systems

n/a	Involved in the study
☐	☒ Antibodies
☒	☐ Eukaryotic cell lines
☒	☐ Palaeontology and archaeology
☐	☒ Animals and other organisms
☐	☒ Human research participants
☒	☐ Clinical data
☒	☐ Dual use research of concern

Methods

n/a	Involved in the study
☒	☐ ChIP-seq
☐	☒ Flow cytometry
☒	☐ MRI-based neuroimaging

Antibodies

Antibodies used

tdTomato (Rockland, 600-401-379, 1:500), manufacturer validated by IF for mouse tissue.
tdTomato (Rockland, 200-101-379, 1:500), manufacturer validated by IF for mouse tissue.
GFP (Invitrogen, A11122, 1:500), manufacturer validated by ICC/IF for HEK-293 cells transfected with H3-GFP construct.
GFP (Rockland, 600-101-215M, 1:500), manufacturer validated by IF for mouse tissue.
GFP (GF090R, nacalai tesque, 04404-84, 1:500), manufacturer validated by IF for mouse tissue.
p21 (Abcam, ab188224, 1:500), manufacturer validated by IHC for mouse tissue.
p21 (Ventana, 760-4453), manufacturer validated by IHC for human tissue.
Ki67 (Abcam, ab15580, 1:200), manufacturer validated by ICC for Mef1 cells.
CK19 (Developmental Studies Hybridoma Bank, TROMA-III, 1:500), manufacturer validated by IHC for mosue tissue.
CK19 (Abbomax, 602-670, 1:500), manufacturer validated by IHC for mosue tissue.
CK19 (Ventana, 760-4281), Zhang Y et al. (2021) validated for human tissue by IHC.
HNF4a (Cell Signalling, 3113s, 1:500), manufacturer validated by IHC for human tissue.
HNF4a (Abcam, ab41898, 1:100), manufacturer validated by IHC for human tissue.
Fah (Abclonal, A13492, 1:500), manufacturer validated for mouse tissue by Western.
β-catenin (BD Pharmingenp, 610153, 1:100), manufacturer validated for A431 cell line by IF.
anti-active-β-catenin (Millipore, Upstate, 05-665, 1:100), manufacturer validated for human tissue line by IHC.
GS (Abcam, Ab49873, 1:1000), Ma R et al. (2020) validated for human tissue by IHC.
E-cadherin (E-cad, 24E10, Cell signaling, 3195, 1:100), manufacturer validated for human tissue line by IHC.
Epcam (Abcam, ab92382, 1:400), Matsumori T et al. (2020) validated for mouse tissue by IF.
Anti-Cytochrome P450 2E1 antibody (CYP2E1, Abcam, ab28146,1:100), manufacturer validated for human cell line by ICC/IF.
OPN (R & D, AF808-SP, 1:500), manufacturer validated for mouse cell line by IF.
A6 (a gift from Valentina Factor, 1:100), Suzuki Y et al (2016) validated for mouse cell line by IF.
Mucin2 (Santa Cruz, sc-15334, 1:400), Kosinsky, RL et al (2015) validated for mouse cell line by IHC.
YAP/TAZ (Cell Signaling, 8418, 1:100), manufacturer validated for human cell line by western.
Sox9 (Millipore, AB5535, 1:1000), manufacturer validated for human tissue by IHC.
GAPDH (Proteintech, 60004-1-Ig, 1:2000), manufacturer validated for human cell line by western.
Donkey anti-rabbit IgG (H+L) Highly Cross-Adsorbed Secondary Antibody, Alexa FluorTM 555 (Thermo fisher scientific, A31572, 1:500), manufacturer validated for detection of rabbit IgG on human cell line by ICC/IF.
Donkey anti-rabbit IgG (H+L) Highly Cross-Adsorbed Secondary Antibody, Alexa FluorTM 488 (Thermo fisher scientific, A21206, 1:500), manufacturer validated for detection of rabbit IgG on human cell line by ICC/IF.
Donkey anti-rabbit IgG (H+L) Highly Cross-Adsorbed Secondary Antibody, Alexa FluorTM 647 (Thermo fisher scientific, A31573, 1:500), manufacturer validated for detection of rabbit IgG on human cell line by ICC/IF.
Donkey anti-Goat IgG (H+L) Highly Cross-Adsorbed Secondary Antibody, Alexa FluorTM 555 (Thermo fisher scientific, A21432, 1:500), manufacturer validated for detection of goat IgG on human cell line by ICC/IF.
Donkey anti-Goat IgG (H+L) Highly Cross-Adsorbed Secondary Antibody, Alexa FluorTM 488 (Thermo fisher scientific, A11055, 1:500), manufacturer validated for detection of goat IgG on human cell line by ICC/IF.
Donkey anti-Goat IgG (H+L) Highly Cross-Adsorbed Secondary Antibody, Alexa FluorTM 647 (Thermo fisher scientific, A21447, 1:500), manufacturer validated for detection of goat IgG on human cell line by ICC/IF.
Donkey anti-Rat IgG (H+L) Highly Cross-Adsorbed Secondary Antibody, Alexa FluorTM 488 (Thermo fisher scientific, A21208, 1:500), manufacturer validated for detection of rat IgG on human cell line by ICC/IF.
Donkey anti-Mouse IgG (H+L) Highly Cross-Adsorbed Secondary Antibody, Alexa FluorTM 647 (Thermo fisher scientific, A31571, 1:500), manufacturer validated for detection of mouse IgG on human cell line by ICC/IF.
Immpress HRP horse anti-rabbit IgG Polymer Detection Kit, Peroxidase (Vector laboratories, MP-7401, 1:1), manufacturer validated for mouse tissue by IHC.
Peroxidase AffiniPure Goat Anti-Rabbit IgG (Jackson ImmunoResearch, 111-035-047, 1:4000), Speckmann T et al (2016) validated for mouse cells by western.
Peroxidase AffiniPure Donkey Anti-Mouse IgG (Jackson ImmunoResearch, 715-035-150, 1:4000), Usman W et al (2018) validated for mouse cells by western.

Validation

Validation statements are included in the statement for "Antibodies used" above.

Animals and other organisms

Policy information about studies involving animals; ARRIVE guidelines recommended for reporting animal research

Laboratory animals	Mice of both male and female at the age of 8-20 weeks were used for experiments with similar aged mice for both control and experimental groups. All mice were maintained on a 129, C57BL6 and ICR mixed background. CK19-CreER, Fah-LSL, R26-tdT, HNF4α-DreER, R26-RL-tdT, CK19-2A-CreER, Rbpjfl/+, R26-NICD-GFP, R26-GFP, R26-Confetti, Ctnnb1fl/+, Ctnnb1lox(ex3)/+ mouse lines were used in this study. All mice were housed at the laboratory Animal center of the Center for Excellence in Molecular Cell Science in a Specific Pathogen Free (SPF) facility with individually ventilated cages. The room has controlled temperature (20-25°C), humidity (30%-70%) and light (12 hour light-dark cycle). Mice were provided ad libitum access to a regular rodent chow diet.
Wild animals	No wild animals were included in this study.
Field-collected samples	No field-collected samples were included in this study.
Ethics oversight	All mice were used in accordance with the guidelines of the Institutional Animal Care and Use Committee (IACUC) of Shanghai Institute of Biochemistry and Cell Biology, Chinese Academy of Sciences. The animal protocol number is SIBCB-S374-1702-001-C1.

Note that full information on the approval of the study protocol must also be provided in the manuscript.

Human research participants

Policy information about studies involving human research participants

Population characteristics	Glass slides with Formalin-fixed paraffin-embedded (FFPE) sections from 70 patient livers were obtained from the University Hospital Basel Tissue Bank in Switzerland. (6 healthy livers, 6 NASH, 6 Hepatitis B, 6 Hepatitis C, 9 Acute Liver Failure, 5 Primary Biliary Cirrhosis (PBC), 5 Primary Sclerosing Cholangitis (PSC), 5 Autoimmune Hepatitis (AIH), 5 ASH Cirrhosis, 6 NASH cirrhosis, 6 Hepatitis B Cirrhosis, 5 Hepatitis C Cirrhosis). All samples were leftover routine diagnostic material and not associated with or derived from a clinical study. Disease indications were diagnosed by 2 pathologists and a hepatologist, who are co-authors of this study. Healthy livers were classified by normal morphology during histopathological assessment. Covariate-relevant population characteristics of the human patients (gender, age) are included in a supplementary table.
Recruitment	The biopsies were originally acquired for routine diagnostic and patients signed a general informed consent for the use of remaining tissue for research purposes in accordance with the Swiss Federal Human Research Act (HRA). Patients did not receive compensation. There was no recruitment for this study and therefore no associated bias since we exclusively used leftover diagnostic material. Samples were selected only by disease pathology and not by any other factors. Further separation of disease groups into subgroups considering age and gender was not performed due to limited numbers of available samples (e.g. since some diseases are more prevalent in males, some more in females). Variability in the data may be associated with differences in covariates or could be due to differences in tissue quality. However, consistent data across different diseases when compared to healthy patients indicates that this did not impact our study.
Ethics oversight	The study was approved by the ethics committee of Northwest and Central Switzerland (EKNZ) as part of the EKNZ (former EKBB:361/12).

Note that full information on the approval of the study protocol must also be provided in the manuscript.

Flow Cytometry

Plots

Confirm that:

- [x] The axis labels state the marker and fluorochrome used (e.g. CD4-FITC).
- [x] The axis scales are clearly visible. Include numbers along axes only for bottom left plot of group (a 'group' is an analysis of identical markers).
- [x] All plots are contour plots with outliers or pseudocolor plots.
- [x] A numerical value for number of cells or percentage (with statistics) is provided.

Methodology

Sample preparation	Liver cells were isolated by standard two-step collagenase perfusion as described previously. The liver was perfused with perfusion medium using a peristaltic pump. Then, the liver was next perfused with medium containing collagenase type I (150U/ml; Invitrogen) for 10 min to adequately digest the liver. After removing the gallbladder, the liver was dissected with cold re-suspension buffer (0.5% BSA and 2mM EDTA in PBS) to free the hepatic cells. Then the cell suspension was passed through a 70 μm cell strainer (BD Biosciences, 352350) and centrifuged at 50 g for 3 min at 4°C. The non-parenchymal cells remained in supernatant were collected and passed through a 40 μm cell strainer (BD Biosciences, 352340) then centrifuged at 400 g for 5 min at 4°C. The cell pellet was re-suspended in red blood cell lysis buffer (eBioscience, 00-4333-57) for 5 min at room temperature and washed with cold re-suspension buffer and centrifuged at 400 g for 5 min. The washing step was repeated once again. Subsequently, cells were stained with the positive selection antibody (anti-mouse Epcam-APC, eBioscience, 17-5791-82) diluted in re-suspension buffer for 30 min in the dark at 4°C. After staining, cells were washed with

	re-suspension buffer and centrifuged at 400 g for 5 min. Epcam+ cells were enriched by using APC microbeads (130-090-855, Miltnyi Biotec) according to the manufacturer's protocols.
Instrument	Sony MA900 Flow Cytometer.
Software	FlowJo software (Tree star).
Cell population abundance	About 1X10^6 non-parenchymal cells were analysis. BECs comprise about 10% of the single cell suspension.
Gating strategy	First, remove small debris in FSC-A verse SSC-A gating. And then doublets were excluded in SSC-A verse SSC-H gating. Dead cells were excluded on DAPI staining. Then BECs population was collected in gates determined on Epcam antibody staining. Gating strategies are shown in Extended Data Fig. 4a.

☒ Tick this box to confirm that a figure exemplifying the gating strategy is provided in the Supplementary Information.
