# Supplementary figures and images for: Bipotent transitional liver progenitor cells contribute to liver regeneration
Source: Nat Genet. 2023 Mar 13;55(4):651–64. doi: 10.1038/s41588-023-01335-9 (PMC10101857; doi:10.1038/s41588-023-01335-9)

FAH

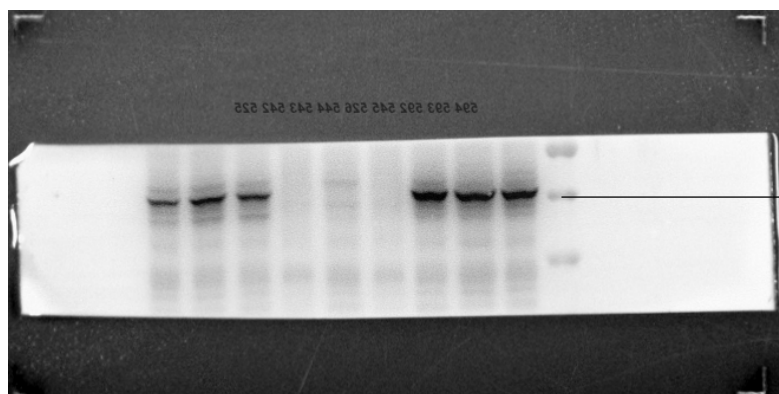

40 kDa

GAPDH

35 kDa

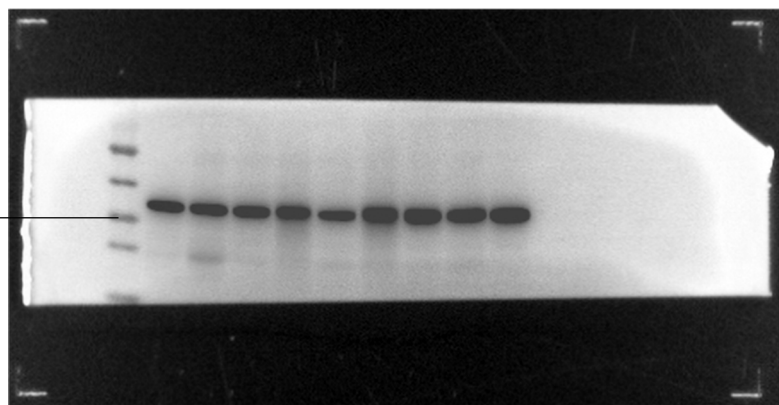

Supplement: Source Data Fig. 1 — Unprocessed western blots for Extended Data Fig. 1d. [file 41588_2023_1335_MOESM3_ESM.pdf]
